# Neanderthal hunting strategies inferred from mortality profiles within the Abric Romaní sequence

**DOI:** 10.1371/journal.pone.0186970

**Published:** 2017-11-22

**Authors:** Juan Marín, Palmira Saladié, Antonio Rodríguez-Hidalgo, Eudald Carbonell

**Affiliations:** 1 Muséum National d'Histoire Naturelle (MNHN), Institut de Paléontologie Humaine (IPH), 1 rue René Panhard, Paris, France; 2 Equipo de Investigación Primeros Pobladores de Extremadura. Casa de la Cultura Rodríguez Moñino, Avd. Cervantes s/n, Cáceres, Spain; 3 IPHES, Institut Català de Paleoecologia Humana i Evolució Social, Unit associated with the Consejo Superior de Investigaciones Científicas (CSIC), Zona Educacional 4, Campus Sescelades URV (Edifici W3), Tarragona, Spain; 4 Área de Prehistoria, Universitat Rovira i Virgili (URV), Avinguda de Catalunya 35, Tarragona, Spain; 5 GQP-CG, Grupo Quaternário e Pré-História do Centro de Geociências (uI and D 73 –FCT) Maçao, Portugal; 6 Unit associated to CSIC. Departamento de Paleobiologia. Museo Nacional d Ciencias Naturales, C/ José Gutierrez Abazcal, Madrid, Spain; 7 Department of Prehistory, Complutense University, Prof. Aranguren s/n, Madrid, Spain; 8 IDEA (Instituto de Evolución en África), Calle Covarrubias 36, Madrid, Spain; Max Planck Institute for the Science of Human History, GERMANY

## Abstract

Ungulate mortality profiles are commonly used to study Neanderthal subsistence strategies. To assess the hunting strategies used by Neanderthals, we studied the ages at death of the cervids and equids found in levels E, H, I, Ja, Jb, K, L and M of the Abric Romaní sequence. These levels date between 43.2 ± 1.1 ka BP (^14^C AMS) and 54.5 ± 1.7 ka BP (U-series). The degree of eruption and development of the teeth and their wear stages were used to determine the ages of these animals at death, and mortality profiles were constructed using these data. The equids display prime dominated profiles in all of the analyzed levels, whereas the cervids display variable profiles. These results suggest that the Neanderthals of Abric Romaní employed both selective and non-selective hunting strategies. The selective strategy focused on the hunting of prime adults and generated prime dominated profiles. On the other hand, non-selective strategies, involved the consumption of animals of variable ages, resulting in catastrophic profiles. It is likely that in the selective hunting events were conducted using selective ambushes in which it was possible to select specific prey animals. On the other hand, encounter hunting or non-selective ambush hunting may have also been used at times, based on the abundances of prey animals and encounter rates. Specific hunting strategies would have been developed accordance with the taxa and the age of the individual to be hunted. The hunting groups most likely employed cooperative hunting techniques, especially in the capture of large animals. Thus, it is not possible to uniquely associate a single mortality profile with the predation tactics of Neanderthals at Abric Romaní.

## Introduction

Mortality profiles are an instrument traditionally used to infer the origins of fossil assemblages in archaeology and paleontology [1–6]. Kurten [4] and Voorhies [7] were pioneers in the study of paleontological assemblages. They applied principles developed by ecologists to fossil records (e.g. [8, 9]), and established the ages at death of animals in order to construct life tables, which they used to infer the population dynamics represented by the fossil record. Mortality profiles were later employed at North American Paleoindian archaeological sites [10–12]. These studies focused on bison kill sites where mass predation events occurred in order to establish hunting seasonality [10–12].

Age at death research has conventionally focused on animal dentition. Non-destructive methods have been developed for these assessments, such as the measurement of the crown heights of teeth [3, 13, 14] and analyses of occlusal surface wear [15–17]. Both methods are based on comparing the assessed tooth wear stage with reference collections that include animals whose ages at death are known. Destructive analysis methods have also been used. Cementochronology is based on counting the layers of cyclically deposited cement, which alternate between relatively thin and relatively thick and reflect a growth periodicity that generally corresponds to an annual cycle [18–20].

Two types of plots are typically used in archaeological studies with this focus: 1) Mortality profiles show the relative or absolute frequency of faunal remains within each age range [3, 5]. 2) Survivorship curves show the surviving individuals in each interval, starting with the total number in the assemblage [3, 5]. Age at death estimates are generally compiled in histograms and linear graphs (e.g. [21, 22]). Greenfield [23] introduced the use of triangular graphs in the analysis of mortality profiles, although the works of Stiner [5, 6] popularized the use of this type of diagram. The distribution of the three age groups (juvenile, prime adult and old adult) can be graphically represented to show the different mortality profiles. This makes it possible to compare the mortality profiles obtained from archaeological assemblages with profiles based on current observations. Two types of mortality profiles are characteristic of the populations of large mammals, and are commonly referred to as catastrophic (or living-structure) profiles and attritional profiles [2, 5, 7, 24]. Catastrophic mortality profiles reflect the ranges of ages that can be observed in living groups of animals, which are present in direct relation to their abundance in the ecosystem. They are produced by ambush predators (e.g. lions, leopards and tigers), by communal hunting events carried out by hominins, and by massive mortality events caused by drowning, famines, etc [2, 5, 7, 25]. Attritional mortality profiles are characterized by a high frequency of younger and older individuals, which are the weakest and most easily hunted animals, and such profiles are generally produced by cursorial hunters (e.g. cheetahs, wolves, spotted hyenas and lycaons) [2].

Using mortality profiles derived from Middle and Upper Paleolithic sites in Italy, Stiner [5] identified a progressive increase in the preference for hunting prime adult animals, an age range not exploited by other predators. This type of prey selection is ecologically complementary to cursorial predators and, to a lesser extent, ambush predators, and it allows different predators to exploit the same prey population while minimizing competition [2, 5, 6, 26]. Stiner [5] defined this hunting behavior of hominin groups as the human predatory niche. In her earliest work, Stiner [5, 6] indicated that the mortality profiles of the early Mousterian (pre-45,000 BP) are characterized by a non-selective pattern in the ages of prey. This pattern is in striking contrast to the selective pattern of the late Mousterian after 40,000 BP [5, 6], in which prime adults are more abundant. In her later work, she rejected differences between the periods mentioned above because she had observed mortality profiles that included the totality of the catastrophic and prime-dominated areas, which, on average, indicate a slight bias toward the selection of prime adults [27]. Finally, she indicated that the presence of profiles containing prime adults during the Middle Paleolithic on different continents suggests the initial development of this behavior in the hunting of large ungulates [28].

Current opinion holds that Neanderthals were skilled hunters of large mammals, and that they had great behavioral flexibility, allowing them to exploit a wide spectrum of resources [5, 27–48]. However, the key component of Neanderthal subsistence was the exploitation of large and medium ungulates [49, 50]. In most of the Mousterian sites of the Mediterranean basin, this group of animals includes deer, horses and aurochs [33, 41–43, 48, 51].

The mortality profiles obtained from different Middle Paleolithic sites indicate that Neanderthal groups generated several types of mortality profiles. These profiles range from selective profiles like that seen at Gabasa (Spain) [52] in which juvenile animals are favored, to catastrophic profiles like that seen at Manie, Madonna and Fate (Italy) [53]. However, numerous sites show a predilection for the hunting of prime adults, as seen at Combe-Grenal (France) [52], in level E of Lazaret (France) [53], in level 7 of Pech-de-l’Aze I (France) [54], in Salzgitter Lebenstedt (Germany) [55], in Grotta Breuil (Italy) [27] and Misliya (Israel) [56], among others. This tendency has also been documented at several Lower Paleolithic sites, such as Wallertheim (Germany) [56], Qesem Cave (Israel) [57], Gran Dolina de Atapuerca (Spain) in levels TD6.2 [58] and TD10.1 [59], Cuesta de la Bajada (Spain) [60] and FLK-Zinj (Tanzania) [61, 62].

The mortality profiles determined from archaeological sites yield information on the hunting skills of the predators that occupied those sites [52]. According to optimal foraging models, hunters (human and non-human) select prey animals that provide high return rates, usually in terms of calories per unit time or per unit energy spent during foraging [63]. The availability of prey animals and the risk involved in hunting them, among other factors, affect the prey selections made by predators. Thus, the abundance of prey animals at an archaeological site provides information on the skills, techniques and hunting strategies of the hunters [52].

Steele and Baker [64] argue that discussions of human predation must include the use of tools, elaborate communication systems, social hunting, cooperation and sharing, the exploitation of large territories, the transport of prey and differing prey consumption. Thus, they established several categories into which hunting events can be classified. These categories are primarily separated in terms of the number of hunted animals and the number of hunters. Taking that proposal as a starting point, Driver [65] studied the social organization and technology of the participants in hunting events. Thus, the number of hunted animals and the way in which they are killed distinguish simple predation events from sequential or mass predation events. The size of the hunter group and its social organization might reflect individual hunters, cooperative groups or communal groups. Each of these types of predation can occur in multiple ways as a function of the tactics (e.g. hunting by driving, ambush or stalking) and techniques used (e.g., spears, bows and arrows, or nets) [64–66].

The goal of this paper is to outline the hunting strategies, including the tactics, developed by the Neanderthals of Abric Romaní over time through the reconstruction of the dental series of the equids and cervids recovered at the site and the determination of their ages at death. Abric Romaní provides the opportunity to study a 15,000-year-long sequence at a Neanderthal site, allowing us to examine the hunting strategies that the Neanderthals employed during their occupation of the rock shelter.

## The Abric Romaní

The archaeological site of Abric Romaní is a rock shelter located in the northeastern portion of the Cinglera del Capelló cliff, 45 km northwest of Barcelona, Spain. The stratigraphy is made up of 20 m of well-stratified travertine sediments. Rock fragmentation and alluvial and biochemical sedimentary processes have generated beds of consolidated stones, gravels, calcarenites and calcilutites interspersed with very fine archaeological levels. Uranium-series and radiocarbon dates place the Abric Romaní deposit between 70 and 40 kyr (Table 1). The sedimentation rate is estimated to have been approximately 0.6 m/kyr [67]. Excluding level A, all of the archaeological levels correspond to the Middle Paleolithic. The Abric Romaní sequence ranges from Marine Isotope Stage (MIS) 4 (sterile levels) to the first half of MIS 3 (archaeological levels). It includes the Dansgaard-Oeschger (D-O) cycles, which extend from 19 to 12, as well as the Heinrich Stadium (HS) from 6 to 5 [68] (Table 1).

**Table 1:** Summary by layer, occupation type, lithics, wood used and previously specified transport strategies [67, 68, 71, 73, 75, 81–84, 88–91, 93–95].

Abric Romaní	Model of occupation	Lithcis		Charcoals, hearths and wood remains	Faunal Taxa		Anthropogenic / canivore modifications	Chronology	D-O HS	MIS
		Raw Material	Knapping Methods		Ungulate (MNI)	Carnivores				
**Level E**	Residential camp	Flint (90%)	Discoid and levallois methods. Lithic tools: Cores (2%) and retouched (4%) tools (denticulates and noches) are scarce; small flakes (41%) and fragment flakes (49%) are most common.	Hearths = 11	*Cervus elaphus* (3)	*Canis lupus* (1)	Anthropogenic modifications: Cut marks 7%; Percussion marks 11%	43.2 ± 1,1 ka BP (^14^C AMS)	12	3
		Limestone (5%)			*Equus ferus* (3)					
		Quartz (3%)			*Bos primigenius* (4)	*Lynx sp*. (1)				
		Others (2%)			*Rupicapra pyrenaica* (4)					
					Proboscidea (1)	*Crocuta crocuta* (1)	Carnivore modifications 0.5%			
**Level H**	Residential camp: Short term occupation	Flint (60%)	Discoid and levallois methods. Lithic tools: Cores (1%) and retouched (4%) tools (denticulates and noches) are scarce; small flakes (49%) and fragment flakes (17%) are most common.	*Pinus sp*. *=* 30.4%	*Cervus elaphus* (3)	*Panthera leo spelaea* (1)	Anthropogenic modifications: Cut marks 2.85%	46.6 ±1.7 ka BP (U/Th)	13/ HS5	
				*Artemisia* = 39.3%				Plat sup: 45.1 ± 3.1 ka BP (U/Th)		
		Limestone (25%)		*Poaceae* = 21.4%	*Equus ferus* (2)					
				Others trees = 3.5%				Plat inf: 46.5 ±1.1 ka BP (U/Th)		
		Quartz (6%)		Hearths = 10	*Rinocerotidae* (1)					
				Preservation of wood is scarce (4 remains: 2 *Pinus*; 1 *Junipeurs*)				44.5 ±1.2 ka (^14^C AMS)		
**Level I**	Residential camp: Short term occupation; Highly mobile group; Linear mobility; Planning of long movement	Flint (50%) outcrops to 5km and 25 km	Discoid method. Lithic tools: Cores (1%) and retouched (2%) tools (denticulates and noches) are scarce; small flakes (37%) and fragment flakes (56%) are most common.	*Pinus silvestris/nigra*	*Cervus elaphus* (7)		Anthropogenic modifications: Cut marks 2.84%; Percussion marks 2.53%	46 ka BP (U/Th)		
		Quartz (26%) and Limestone (21%) local 5km.		*salzmannii*. = 60%	*Equus ferus* (7)		Carnivore modifications 1.5%	Plat sup: 45.1 ± 3.1 ka BP (U/Th)		
				Hearths = 16	*Bos primigenius* (1)			Palt inf: 48.6 ± 2.3 ka BP (U/Th)		
				Preservation of wood is scarce, fuel wood accumulations.						
**Level J**	Residential camp: Long tern occupation.	Flint (75%) outcrops to 5km and 25 km Quartz (11%) and Limestone (12%) local 5km	Discoid and Centripetal methods. Lithics tools: Denticulates (3%) and cores (1%) are scarce; small flakes (36%) and fragment flakes (50%) are most common.	*Pynus sylvestris* = 71%	*Cervus elaphus* (11)		Anthropogenic modifications: Cut marks 11%; Percussion marks 20%	Plat sup: 49.3 ± 1.6 ka BP (U-series)		
				*Pinus uncinata =* 3.4%	*Equus ferus/hydruntinus*(21)			Plat inf: 50.4 ± 1.6 ka BP (U-series)		
				Hearths = 60	*Stephanorhinus hemitoechus* (5)					
				Preservation of wood is scarce, fuel wood accumulations. Pointed wooden element.	*Bos primigenius* (7)		Carnivore modifications 1%	47 ± 2.1 ka (^14^C AMS)		
					*Rupicapra pyrenaica* (4)					
**Level K**	Residential camp: Short term occupation	Local and semi-local raw materials between 15 and 20 km	Discoid method. Lithics tools: Denticulates (2%) and cores (0.7%) are scarce; small flakes (30%) and fragment flakes (34%) are most common.	*Pynus sylvestris* = 54.51%	*Cervus elaphus* (11)		Anthropogenic modifications: Cut marks 4.1%; Percussion marks 10.7%	Plat sup: 50 ± 1.6 ka BP; (U-series)	14	
		Flint (47.9%)		*Pinus uncinata =* 0.4%	*Equus ferus* (8)					
		Limestone (19.2%)		Hearths = 25	*Bos primigenius* (1)		Carnivore modifications 3%	Plat inf: 51 ± 9 ka BP (U-series)		
		Quartz (28%)		Little accumulation of negative with signs of cremation						
**Level L**	Residential camp: Short term occupation	Local and semi-local raw materials between 15 and 20 km	Discoid method. Lithics tools: Denticulates (2.7%) and cores (1.4%) are scarce; small flakes (46.2%) and fragment flakes (39.8%) are most common.	*Pynus sylvestris* = 63.78%	*Cervus elaphus* (7)		Anthropogenic modifications: Cut marks 6.7%; Percussion marks 3.4%	52.5 ± 1 ka BP (U-series)		
		Flint (83.7%)		*Pinus uncinata =* 2.8%	*Equus ferus* (2)					
		Limestone (9.9%)		Hearths = 23			Carnivore modifications 0.8%			
		Quartz (3.3%)		Four accumulations grouped together to hearths	*Bos primigenius* (4)					
**Level M**	Residential camp: Long tern occupation.	Local and semi-local raw materials between 10 and 30 km	Discoid and centripetal methods. Lithics tools: Denticulates (0.1%) and cores (0.02%) are scarce; small flakes (47.65%) and fragment flakes (31.25%) are most common.	*Pinus sylvestris/nigra =* 59%	*Cervus elaphus* (8)	*Ursus sp*. (1)	Anthropogenic modifications: Cut marks 6.7%; Percussion marks 3.7%	54.5 ± 1.7 ka BP (U-series)		
		Flint (80%)		*Pinus uncinata* = 1.5%	*Equus ferus* (4)	*Crocuta crocuta* (1)				
		Limestone (9.4%)		Hearths = 37	*Stephanorhinus hemitoechus* (1)	*Felis silvestris* (1)	Carnivore modifications 0.3%			
		Quartz (5.3%)		Four accumulations grouped together to hearths	*Bos primigenius* (4)					

The site was discovered in 1909 by Amador Romaní and excavated during different periods throughout the 20th century. The current intervention, in which the full extent of the rock shelter (c. 300 m^2^) has been excavated, began in 1983. Numerous well-preserved combustion structures have been identified [69–71] in addition to wood remains, including negatives and carbonized positives [72–75].

The lithic record is typical of Middle Paleolithic assemblages. Flakes predominate, whereas cores and retouched flakes are scarce [76–81] (Table 1). Most of the raw material is derived from local sources (flint, quartz and limestone) (Table 1) [81–85].

Faunal remains are very abundant in all levels of Abric Romaní. In total, 38,228 faunal remains belonging to 13 different taxa have been recovered. Among the ungulates, cervids (*Cervus elaphus*) and equids (*Equus ferus/Equus hydruntinus*) are the most abundant animals, according to the number of identified specimens (NISP), the minimum number of elements (MNE) and the minimum number of individuals (MNI). The remains of aurochs (*Bos primigenius*) are also common in the lower levels (I, J, K, L, M, O and P), and the chamois (*Rupicapra pyrenaica*) is common in the upper levels (A, B, D, E, F, G, H and J). The remains of rhinoceros (*Stephanorhinos cf*. *hemitoechus*) have been documented in levels H, J and M, although they are scarce. A proboscid femur was recovered from level E (Table 1). Zooarchaeological studies of each level indicate that the faunal record is the result of Neanderthal activity, which was characterized by primary access to animal carcasses and complete exploitation of their resources [86–89]. By means of the density of the faunal remains, as well as taphonomic analysis and anatomical refitting, it has been possible to establish areas where specific activities were performed. These analyses, along with the characterization of hearths, have resulted in the identification of sleeping areas, cleaning zones and animal processing areas in some of the levels [71, 86–93]. In all the levels, all of the activities associated with animal butchering have been documented, indicating the complete exploitation of animal resources. In addition, numerous thermoalterations have been documented. Evidence of carnivore activity is very scarce and is thought to have resulted from scavenging of the remains left by human groups [71, 73, 79, 81, 82, 86, 88–91, 94]. The faunal assemblage reflects the highly variable transport of animal carcasses. This transport ranged from complete animals to just a few elements. In general, anatomical elements that were high in unsaturated fat were preferentially transported to the rock shelter [94].

The occupation types that produced the archaeological record can be separated into two groups, long-term and short-term (and/or non-residential) occupation events [71, 73, 81, 82, 90–93, 95] (Table 1). In both of these models, all of the hearths were reused, especially during the long-term occupation events, and these structures were therefore preserved. Therefore, the materials present within the Abric Romaní correspond to the superposition of different occupational events [71, 92].

## Materials and methods

### Materials

To determine the mortality profiles of the equids and cervids found in the Abric Romaní sequence, 486 teeth from levels E, H, I, Ja, Jb, K, L and M were examined. Of these teeth, 259 were derived from equids, and 227 from cervids. The proportions of maxillary and mandible teeth, 126:133 for the equids and 101:126 for the cervids, are very similar for both groups of animals. A large fraction of the dental remains was found isolated within the site. In some cases, fragments of maxillae and mandibles were recovered with teeth anchored in their alveoli (62 dental remains in total). The number of analyzed teeth found in each level and their integrity (in situ or isolated teeth) is reported in Table 2. To establish dental age, various methods were combined depending on the type of tooth (superior or inferior and anterior or jugal) and the taxon being considered. All materials used in this study are deposited at the Institut Català de Paleoecologia Humana i Evolució Social (IPHES). No permits were required for the described study, which complied with all relevant regulations.

**Table 2:** Number of teeth analyzed in terms of archaeological level, species and integrity (isolated or within dental series).

Layer	Taxa	Integrity	N	Total
E	Equids	Isolated tooth	10	10
		Tooth in bone	0	
	Cervids	Isolated tooth	2	6
		Tooth in bone	4	
H	Equids	Isolated tooth	5	9
		Tooth in bone	4	
	Cervids	Isolated tooth	4	7
		Tooth in bone	3	
I	Equids	Isolated tooth	33	39
		Tooth in bone	6	
	Cervids	Isolated tooth	3	39
		Tooth in bone	36	
Ja	Equids	Isolated tooth	77	113
		Tooth in bone	36	
	Cervids	Isolated tooth	26	45
		Tooth in bone	19	
Jb	Equids	Isolated tooth	26	26
		Tooth in bone	0	
	Cervids	Isolated tooth	1	1
		Tooth in bone	0	
K	Equids	Isolated tooth	15	19
		Tooth in bone	4	
	Cervids	Isolated tooth	20	58
		Tooth in bone	38	
L	Equids	Isolated tooth	10	10
		Tooth in bone	0	
	Cervids	Isolated tooth	5	20
		Tooth in bone	15	
M	Equids	Isolated tooth	27	33
		Tooth in bone	6	
	Cervids	Isolated tooth	25	51
		Tooth in bone	26	

### Determination of dental age

**Fig 1:**
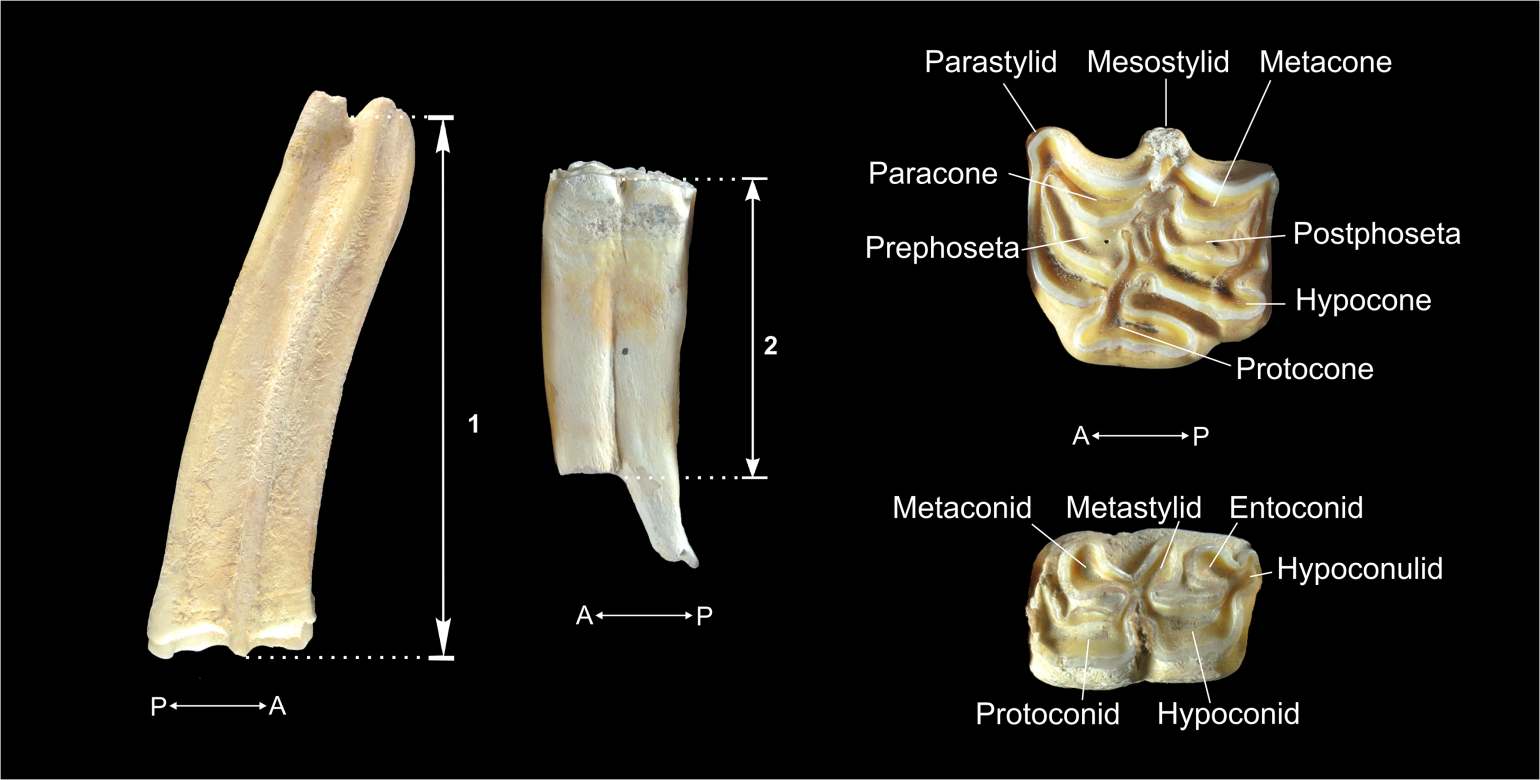
Crown height (CH) (left) and nomenclature of the cheek teeth (right) of equids. 1 = CH of a superior tooth; 2 = CH of an inferior tooth.

In the case of the equids, we looked at the eruption of deciduous teeth and their replacement by permanent teeth [14]. We used the nomenclature for the anatomical descriptions and for assessing the orientation of the jugal teeth established by Levine [14]. The dental ages of permanent superior and inferior premolars and molars with completely worn occlusal surfaces were calculated based on their crown heights. To estimate age, the parameters of a third-order polynomial regression that relates crown height to age were used [13]. The relevant equation is $AGE=\sum_{k=0}^{3}a_{k}\cdot {\left(crown\;height\right)}^{k}$; were a_k_ is the regression coefficient [13, 14, 96]. The data used in the calculation of the polynomial regression are those provided by Fernandez and Legendre [13]. In keeping with the recommendations of Fernandez et al. [22], we estimated the individual average, minimum and maximum ages as a function of the minimum error associated with each prediction equation (E) [13, 22, 96]. Crown heights were measured from the cementum-enamel junction to the highest point of the occlusal surface along the labial face (Fig 1).

**Fig 2:**
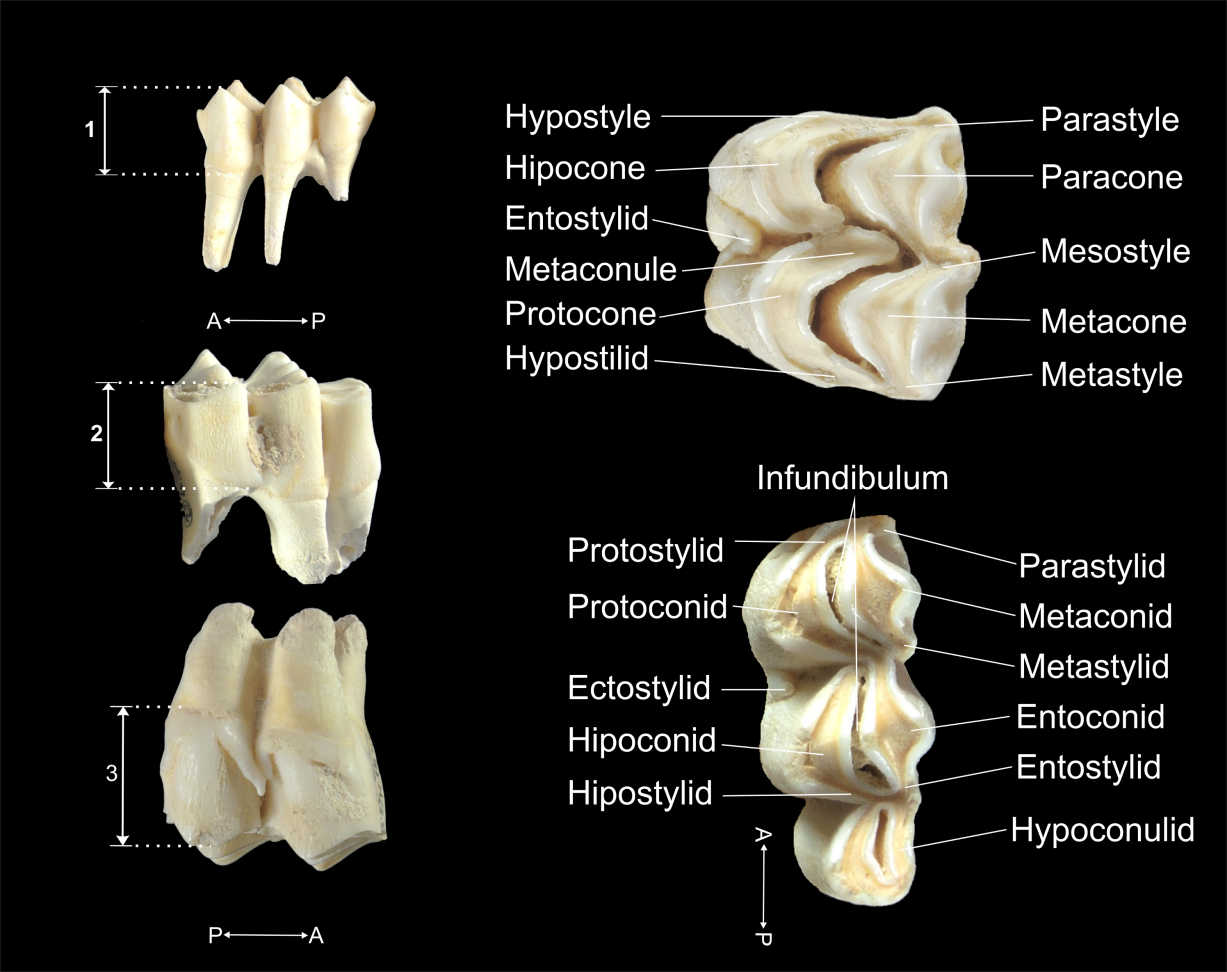
Crown height (CH) (left) and nomenclature of the maxillae and mandible teeth (right) of cervids. 1 = CH of a fourth deciduous premolar; 2 = CH of a third inferior molar; 3 = CH of a second superior molar.

To estimate the dental ages of the cervids, the eruption, replacement and wear of the jugal teeth were analyzed [15]. The anatomical nomenclature of the teeth, the designation of the worn facets and the orientations of the teeth were based on the criteria given by Brown and Chapman [15] (Fig 2). The wear stage codes established by Mariezkurrena [97] and Azorit [98] were also used. These codes were modified for *Cervus elaphus* from Payne’s [16] original work with sheep and goats. To estimate the degree of wear of the maxillary dentition, an approximation was made by analogy to the attrition described by Brown and Chapman [15] for mandibular dentition, assuming that the degree of wear for maxillae is similar [99]. As a complement, the quadratic crown height method (QCHM) was applied to the mandibular and maxillary teeth (dP_4_, M_1_, M_2_ and M_3_ / dP^4^, M^1^, M^2^ and M^3^) to estimate dental age. The crown height was measured on the labial faces of the lower teeth and on the lingual faces of the upper teeth, on the anterior lobe between the occlusal surface and the cement-enamel junction [3, 99, 100] (Fig 2). The prediction equations applied were: $AGE=AGESes{\left(\frac{CH-{CH}_{0}}{{CH}_{0}}\right)}^{2}$ for the fourth deciduous premolar (dP_4_/^4^) and $AGE=(AGEpel-AGEe){\left(\frac{CH-{CH}_{0}}{{CH}_{0}}\right)}^{2}+AGEe$ for the permanent molars (M_1_/^1^, M_2_/^2^ and M_3_/^3^). Here, *CH* is the crown height of a tooth in mm; *Ch*_*0*_ is the crown height of an unworn tooth in mm; *AGEes* is the age in months, in which dP_4_/^4^ is replaced by P_4_/^4^; *AGEe* is the eruption age in months of M_1_/^1^, M_2_/^2^ and M_3_/^3^; *AGEpel* is the potential ecological longevity (PEL) in months.

### Estimation of the minimum number of individuals and the construction of age groups and size-weight categories

The MNE [101] for mandibles and maxillae was calculated by means of the construction of dental series, taking the side the tooth originated from (left and right) into account and estimating dental age by means of a combination of the methods described above. The sum of isolated teeth and those found anchored in alveoli with known ages provided a fairly complete dental series. Calculating the MNE can facilitate the estimation of MNI [102, 103] for each of the taxa and levels studied.

Individuals were assigned to age groups based on Bunn and Pickering’s [61] method, which uses the potential ecological longevity (PEL), and is a modification of the model described by Stiner [5]. These authors suggest dividing the PEL into five groups: young juveniles and subadult juveniles (<20% PEL), early prime adults (20–50% PEL), late prime adults (50–75% PEL) and old adults (75%>PEL). This PEL sequence is intended to correctly establish the vulnerability of the ungulates to predators, which is not clearly predicted using the age classes established by Stiner [5]. The early and late prime adult individuals are physically robust, difficult to hunt or even dangerous, whereas the young juveniles, subadult juveniles and old adults are physically weak, more vulnerable and easily hunted. These authors restructure the limits between age groups through the progression of wear stages. Thus, the event that separates young juveniles from subadult juveniles is the eruption of M_1_ and M_2_. They note that, unless extraordinary preservation conditions exist, young juveniles will be excluded from the analyses. The boundary between late prime adults and old adults is the loss of the mesial infundibulum of M1 in class 3 bovids (113–340 kg) and the loss of the mesial and distal infundibulum of M2 in class 1 and 2 bovids (4.5–22.5 kg and 22.5–113 kg), which occurs at a PEL of approximately 75%. However, the method proposed by Bunn and Pickering [61] focused on bovids. That proposal has been slightly modified in our work to adapt the old adult PEL to the taxa that are most frequently encountered at the Abric Romaní.

In this work, the PEL used for horses is 300 months (25 years) [35, 104] and the PEL used for cervids is 192 months (16 years) [100].

The following age groups were established for the equids: 1) Young juveniles (12 months = 4% PEL) are identified by the presence of complete deciduous dentition, which erupts between 30–40 days after birth, until the eruption of M_1_. 2) Subadult juveniles (60 months = 20% PEL) are characterized by wear on M1 and limited by the total loss of deciduous dentition and the eruption and initial wear of P4 and M3 (Levine, 1983). Finally, individuals were assigned to the last three groups, 3) early prime adults (144 months = 50% PEL), 4) late prime adult (225 months = 75% PEL), and 5) old adults (more than 225 months = 75%≥ PEL) by measuring the crown heights of the teeth and assigning them to age groups as a function of the percentage of the PEL [14, 22, 96] (Table 3).

**Table 3:** Reference values for the age groups and crown heights used for the calculation of ages.

Age groups	PEL Equids	Crown height (mm)											
		Mandible						Maxilla					
		P_2_	P_3_	P_4_	M_1_	M_2_	M_3_	P^2^	P^3^	P^4^	M^1^	M^2^	M^3^
Early prime	60–144 month	55–28	78–39	79–42	72–38	76–44	78–46	65–32	76–40	81–41	73–40	80–42	82–37
Late prime	144>225 month	28–8	39–20	42–28	38–21	44–23	46–23	32–14	40–22	41–27	40–24	42–27	37–24
Old	225≥ month	8–4	20–10	28–10	21–10	23–10	23–10	14–5	22–10	27–10	24–10	27–10	24–10

The cervids were divided into the following groups. 1) Young juveniles (5 months = 2.6% PEL) are identified by the presence of all deciduous dentition, which is present from birth, to the eruption and wear of M_1_. 2) Subadult juveniles (30 months = 20% PEL) are identified by the first wear on the mesial peak of M_1_ to the start of wear on the distal peak of M_2_ and the end of wear on dP_2_, dP_3_ and dP_4_. 3) Early prime adults (78 months = 50% PEL) are identified by the first stage of wear of the premolars and M_3_, which occurs in *Cervus elaphus hispanicus* around 30–31 months of age, to the disappearance of the infundibulum of M_1_. 4) Late prime adults (144 months = 75% PEL) are distinguished by the disappearance of the infundibulum of M_1_ and the appearance of the line of crown of M_3_, and are limited by the attrition of the mesial and distal infundibulum of M_2_ and the hypoconulid of M_3_. 5) Old adults (~12 years = 75%≥ PEL) are identified by the loss of the mesial and distal infundibulum of M_2_ and the hypoconulid of M_3_, at approximately 144 months [15, 97, 98]. At this time, the fecundity and body fat of females decreases enormously, and the males are not able to defend their harems due to a decline in their physical strength [105–106].

The two most common weight categories at Abric Romaní are large (equids) and medium-sized (cervids). However, the variation in the weight and size of animals over the course of their lives should not be ignored; taxa and weight categories are not equivalent. These variations in animal weight and size are the results of physiological changes that occur during the growth phase, which ends with sexual maturity and implies major changes in the social behavior of animals [35, 106–109].

In the case of equids, the weight of a two-year-old is equivalent to 75% of that of an adult individual, which is ultimately achieved at five years of age [108], the time at which sexual maturity is also reached by both males and females [35, 104]. Therefore, the dividing line between subadult juveniles and prime adults is considered to correspond to the dividing line between medium and large individuals. The social organization of equids is highly hierarchical. A harem is made up of one stallion and several females with their foals. At around two years of age, the male foals abandon their maternal groups to join groups of single males. Unlike males, subadult females are not forced to leave their maternal group, but they often join other harems after two years [108]. Although this behavior highly influences the composition of social groups, individuals older than two years of age have not yet reached the weight or size of an adult individual. Since the prey choices made by predators, including hominins, are primarily based on feed return rates and the difficulty of capture [63, 110, 111], the fact that male juvenile horses leave their maternal groups and join groups of single males does not necessarily change the way they are considered by predators. Therefore, two-year-old individuals are still considered subadult juvenile individuals.

The size of *Cervus elaphus* is especially variable within single populations and between populations found in different environments. Individuals usually continue to grow until they have reached seven years of age. In *Cervus elaphus*, the boundary between subadult juveniles and prime adults (30 months) coincides with two important ethological events: the abandonment of the family group by young males, and the sexual maturity of females [104]. In *Cervus elaphus* sexual maturity also corresponds to the time at which they reach their full body weight [112]. Therefore, the dividing line between subadult juveniles and prime adults is considered to correspond to the dividing line between small and medium-sized individuals. As indicated above, significant fluctuations in the size of this species have been noted within the fossil record [113, 114]. At present, it has been observed that *Cervus elaphus* in poor habitats are smaller. For example, adult males in northern France weight between 120 and 250 kg (150 kg on average) and females weight between 67 and 100 kg (80 kg on average) [115]. On the other hand, on the Iberian Peninsula, males can reach a body weight of approximately 160 kg, whereas females can reach a body weight of approximately 100 kg [106].

### Mortality analyses

Once the age profiles had been obtained, we analyzed the profiles using triangular diagrams [5]. The individuals were assigned to three age groups (young, prime and old), and the proportion of each class was plotted on a triangular graph. To this end, the five groups were sorted into the three age categories mentioned above; Table 4 shows the correspondence between the age groups. Within the triangular graphs, the upper corner represents 100% old adults, the lower right corner indicates 100% prime adults, and the lower left corner indicates 100% young individuals. The areas representing catastrophic and attritional age structures, which occur to the left of the area indicating the dominance of prime adult and to the right of the area indicating the dominance of juveniles, respectively, are indicated on the graph. When a sample is plotted within a triangular graph, its position within one of these five zones is assumed to indicate a mortality profile [2, 5, 21, 99].

**Table 4:** Age group divisions based on Stiner [5] and Bunn and Pickering [61], and the estimated age of cervids and equids.

Age groups [5]	Age groups [61]	Cervids	Equids
Young	Young juvenile	0–5 month	0-10/12 month
	Subadult juvenile	5–30 month	10/12-60 month
Prime adult	Early prime	30–78 month	60–144 month
	Late prime	78–144 month	144–225 month
Old adult	Old	≤144 month	≤225 month

## Results

The MNI was determined to be 97 across the analyzed levels. Of these individuals, 47 are equids and 50 are cervids (Table 5).

**Table 5:** MNE, MNI and MNI by age group for each level of Abric Romaní.

Level	Taxa	MNE		MNI	Juvenile	Prime adult	Old adult
		Mandible	Maxilla				
E	Equids	2	3	3	1	2	
	Cervids	2	1	3	1	2	
H	Equids	1	2	2		2	
	Cervids		3	3		3	
I	Equids	6	6	7	1	6	
	Cervids	7	4	7	5	1	1
Ja	Equids	17	12	15	4	10	1
	Cervids	7	10	10	5	5	
Jb	Equids	7	5	6	1	5	
	Cervids		1	1		1	
K	Equids	6	3	8		7	1
	Cervids	13	5	11	4	5	2
L	Equids	4	1	2		2	
	Cervids	7	1	7	2	4	1
M	Equids	4	2	4	1	3	
	Cervids	9	9	8	3	4	1
Total		92	68	97	12	41	8

### Equids

**Fig 3:**
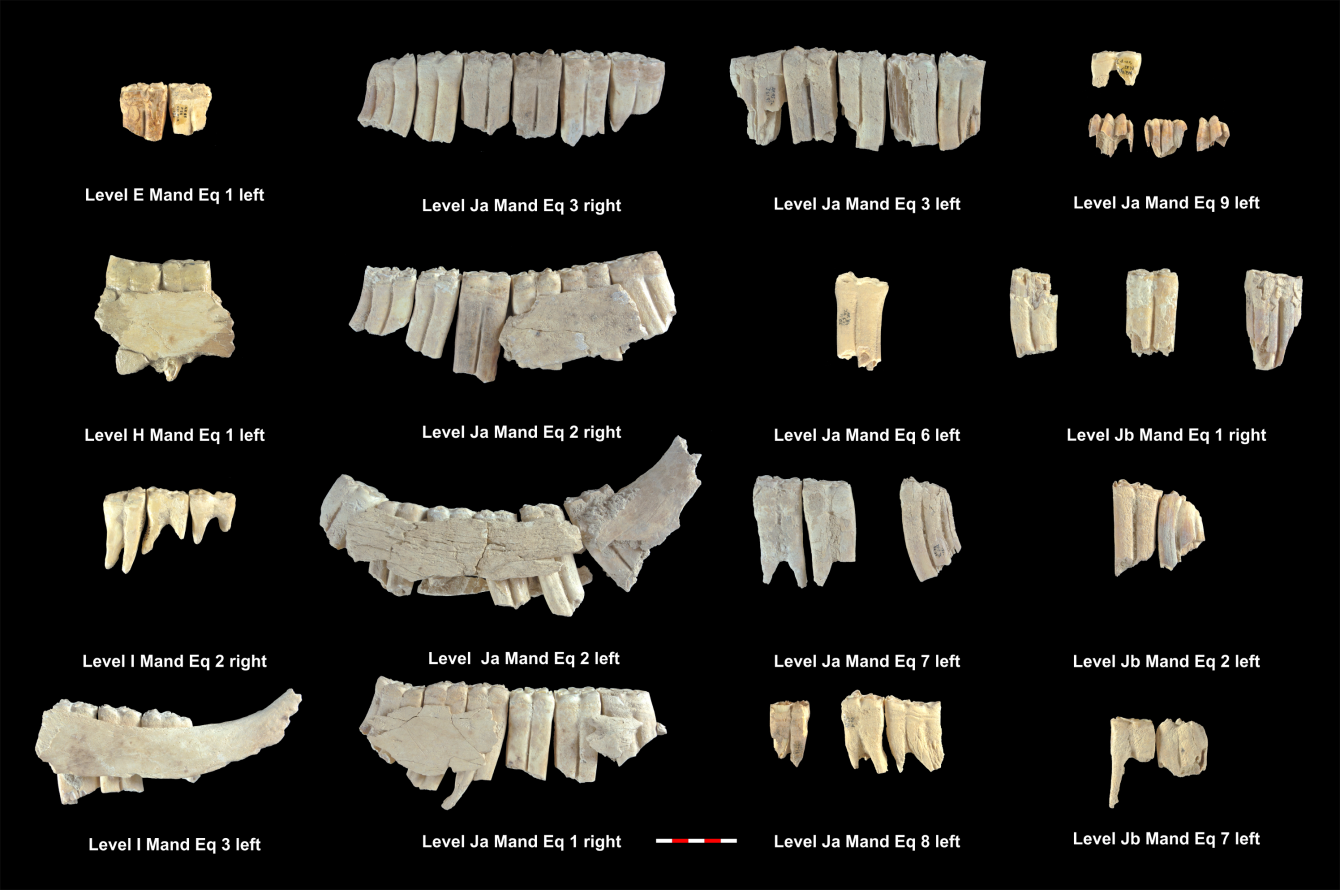
MNI of equids mandibles from levels E, H, I, Ja and Jb. Under each dental series are references to level, individual and side as shown in Table 6.

**Fig 4:**
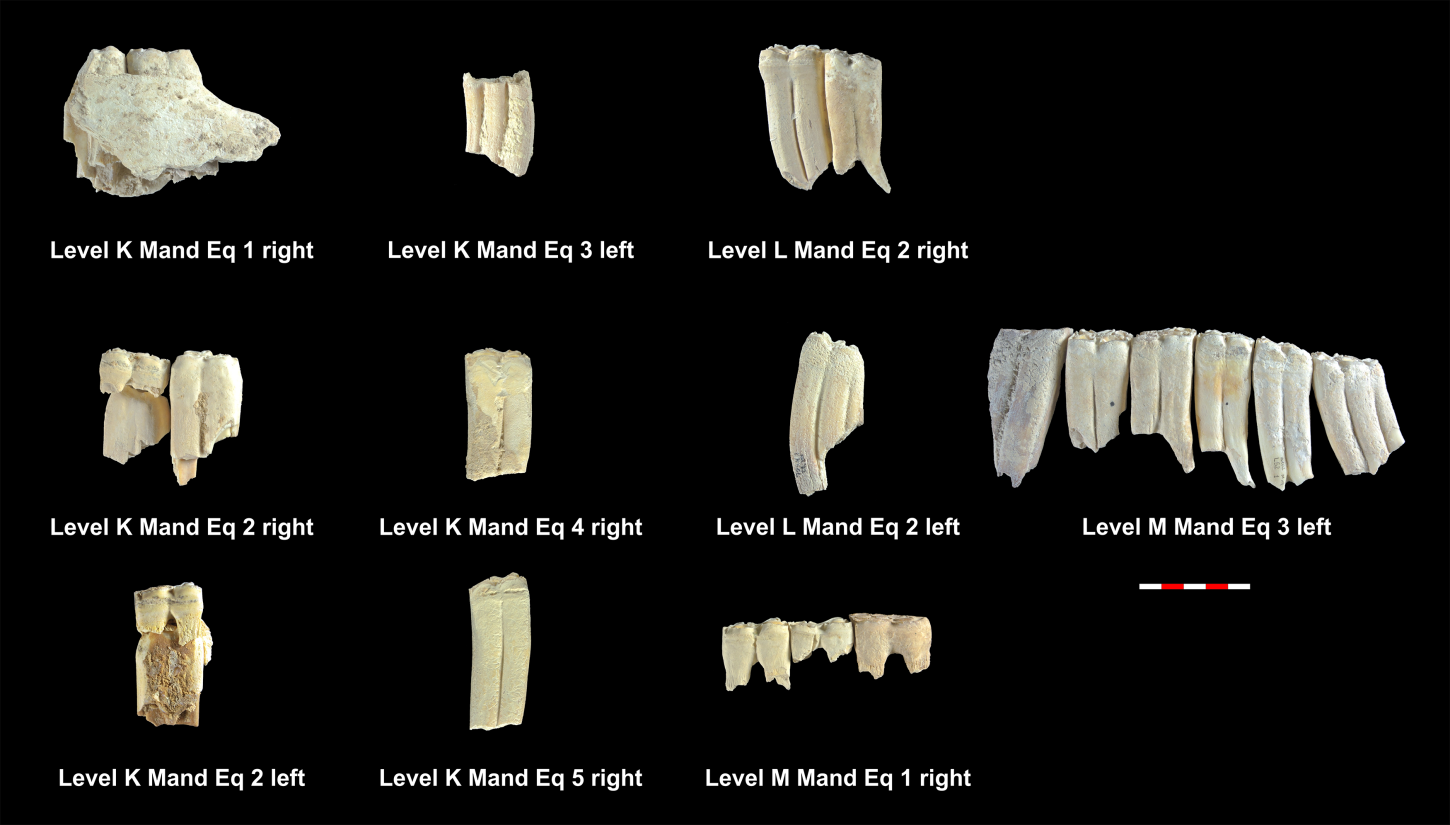
MNI of equids mandibles from levels K, L and M. Under each dental series are references to level, individual and side as shown in Table 6.

**Fig 5:**
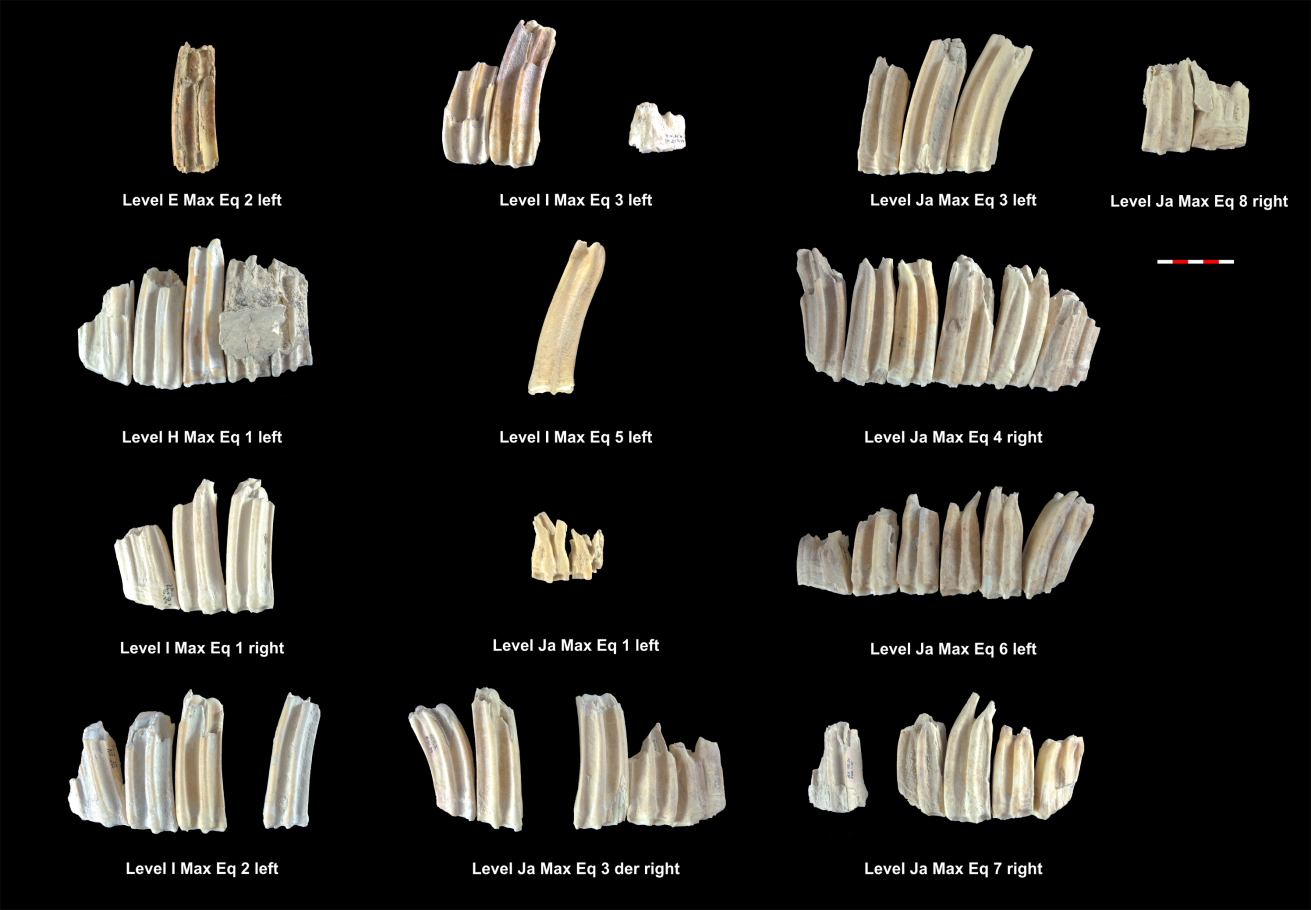
MNI of equids maxillae from levels E, H, I and Ja. Under each dental series are references to level, individual and side as shown in Table 7.

**Fig 6:**
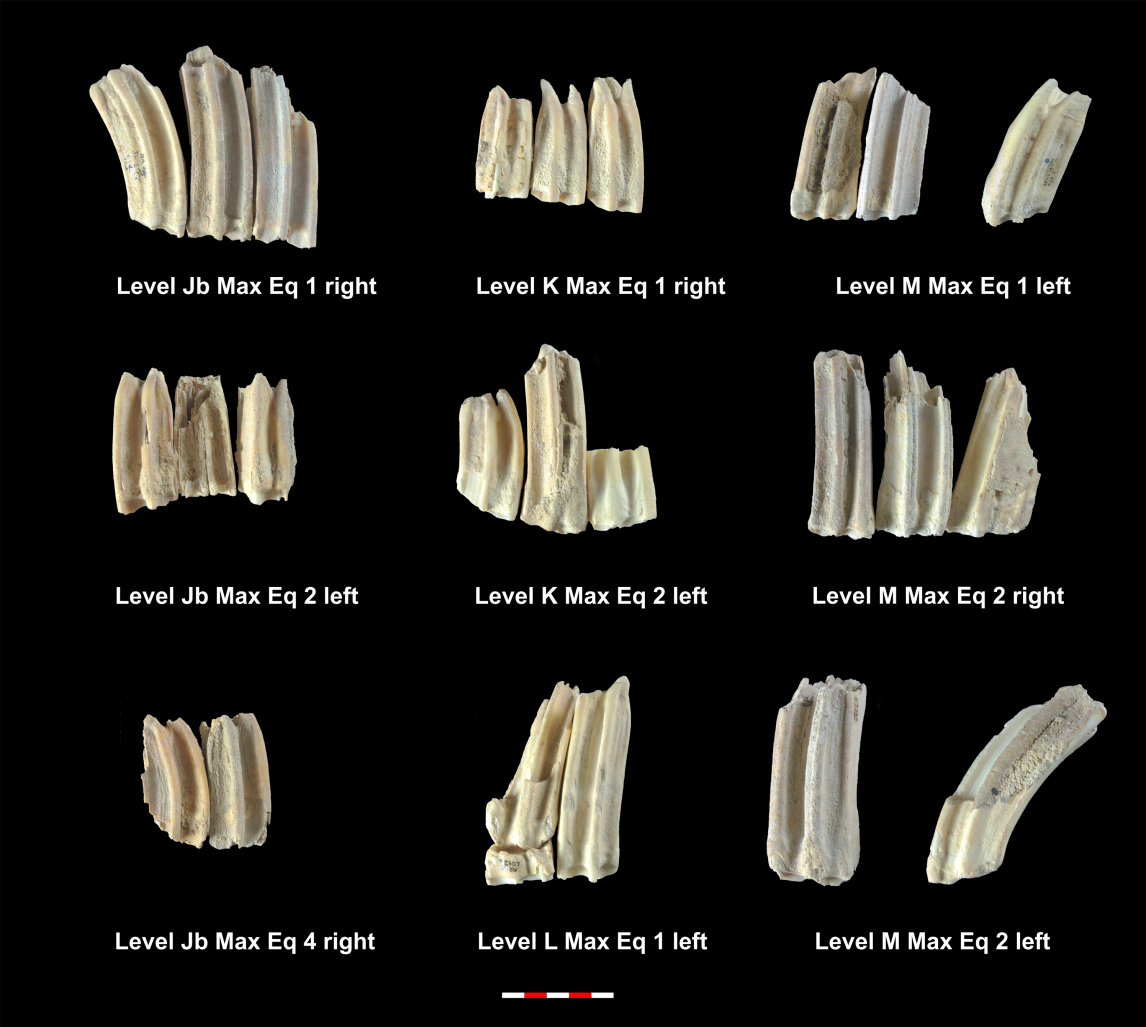
MNI of equids maxillae from levels Jb, K, L and M. Under each dental series are references to level, individual and side as shown in Table 7.

Based on the MNI, the level with the highest number of individuals identified is level Ja with 15 individuals, followed by levels K, I, Jb, M, E, H and L, which contain 8, 7, 6, 4, 3, 2 and 2 identified individuals (Table 5). Tables 6 and 7 show the MNE of mandibles and maxillae. Figs 3–6 show the MNI of the equids by level (S1 Table). We were able to measure the crown heights of 33 mandible teeth and 54 maxillary teeth belonging to a total of 43 individuals, which allowed us to calculate the ages of these animals (Tables 6 and 7).

**Table 6:** Number of equids mandibles at Abric Romaní, indicating level, archaeological reference, MNE, size, dental series, crown height of teeth, age group according to Stiner [5] and Bunn and Pickering [61], and mean, minimum and maximum age in months.

Level	Reference	Individual	Side	Dental series	Crown Height (mm)						Age group		Crown Height Method (Age months)		
					dp_2_/P_2_	dp_3_/P_3_	dp_4_/P_4_	M_1_	M_2_	M_3_	[5]	[61]	Mean Age	Minimum Age	Maximum Age
E	AR85 CIII U51 10/P58 182/AR 98 N42 1	Mand. Eq 1	Left	P3 M3 M2							Prime Adult	Early Prime			
E	AR92 T48 131/P42 19	Mand. Eq 2	Right	dp3 dp4		29.28					Juvenile	Subadult Juvenile			
H	AR 90 2.25 P49 24	Mand. Eq 1	Left	P2 P3	43.61	61.59					Prime Adult	Early Prime	87.84	75.24	100.44
I	AR92 N52 2 / P49 12	Mand. Eq 1	Left	M2 M3							Prime Adult	Early Prime			
I	AR91 P49 13/K55 1/L53 1/L49 6	Mand. Eq 2	Right	M3 dp2 dp3 dp4	16.27	20.36	34.49				Juvenile	Subadult Juvenile			
I	AR 91 R30 5	Mand. Eq 3	Left	M1 M2 M3							Prime Adult	Early Prime			
I	AR 91 M49-R S/C /G60 S/C /M46 2	Mand. Eq 4	Right	P3 P4 M1 M3							Prime Adult	Early Prime			
I	AR 91 CIII S50 8/S50 9/J49 12	Mand. Eq 4	Left	P4 P3 M3							Prime Adult	Early Prime			
I	AR 91 CIII Q51 9	Mand. Eq 5	Left	P3							Prime Adult	Early Prime			
Ja	AR94 O48 35/I64 14/L56 89/L55 27/L58 44	Mand. Eq 1	Left	P2 P3 M1 M2 M3							Prime Adult	Early Prime			
Ja	AR94 K57 106/K57 81/K57 94/ L48 59	Mand. Eq 1	Right	P2 P3 P4 M1 M2 M3				56.17			Prime Adult	Early Prime	81.6	61.8	101.4
Ja	AR94 L51 70/ AR95 J63 4	Mand. Eq 10	Left	M2 M3 M1					34.69	31.66	Prime Adult	Late Prime	153.48	141.36	165.72
Ja	AR95 I49 110	Mand. Eq 11	Right	dp4			31.36				Juvenile	Subadult Juvenile			
Ja	AR94 N48 106/AR 93/ N44 66/AR95 K63 11	Mand. Eq 2	Right	P2 P3 P4 M1 M2 M3							Prime Adult	Early Prime			
Ja	AR93 M46 112/AR94 M48 39/AR92 M58 7	Mand. Eq 2	Left	P2 P3 P4 M1 M2 M3							Prime Adult	Early Prime			
Ja	AR95 J61 46/AR94 L55 2/AR93 J58 51/AR94	Mand. Eq 3	Right	P2 P3 P4 M1 M2 M3	36.83	49.02					Prime Adult	Early Prime	99.36	86.76	111.96
Ja	AR93 P46 1/P48 53/N50 133/AR94 K56	Mand. Eq 3	Left	M1							Prime Adult	Early Prime			
Ja	AR95 M49 80/AR93 L57 16/AR94 K59 60	Mand. Eq 4	Right	M1 P4 M2 M3			51.44		71.45		Prime Adult	Early Prime	78.24	69.36	87
Ja	AR95 R48 54/AR94 L48 23/AR94 K58 61	Mand. Eq 4	Left	P2 P3 M1 M2	33.51						Prime Adult	Early Prime	105.36	90.12	120.06
Ja	AR95 M49 80	Mand. Eq 5	Right	M1							Prime Adult	Early Prime			
Ja	AR94 O48 59/AR 96 L41 6/AR95 J62 36	Mand. Eq 5	Left	P4 M1 M2 M3							Prime Adult	Early Prime			
Ja	AR94 J58 18	Mand. Eq 6	Left	M1							Juvenile	Subadult Juvenile			
Ja	AR93 P47 3	Mand. Eq 7	Right	P4 M1			47.26	45.77			Prime Adult	Early Prime	101.04	75	127.08
Ja	AR94 J51 21/AR91 H53 3	Mand. Eq 7	Left	M3							Prime Adult	Early Prime			
Ja	AR93 M55 1	Mand. Eq 8	Left	dp2 dp3 dp4 M1	11.42	10.64					Juvenile	Subadult Juvenile			
Ja	AR92 plat sup /AR95 P51 12/ AR95 J65 3	Mand. Eq 9	Left	P3		11.79					Juvenile	Subadult Juvenile			
Jb	AR95 O52 36/N49 1/AR99 M41 107	Mand. Eq 1	Right	P2 M2 M1							Juvenile	Subadult Juvenile			
Jb	AR95 M54 130/D46 1/M57 15	Mand. Eq 2	Left	P2 M2 M3							Juvenile	Subadult Juvenile			
Jb	AR95 M54 184/O53 3/M57 20	Mand. Eq 3	Right	P2 M2 P3							Prime Adult	Early Prime			
Jb	AR95 M51 107/O55 31	Mand. Eq 3	Left	M2 M3							Prime Adult	Early Prime			
Jb	AR95 O51 14	Mand. Eq 4	Left	M2											
Jb	AR95 L54 27/M54 173	Mand. Eq 5	Left	M1 M2											
Jb	AR95 N53 79/AR99 M42 19	Mand. Eq 7	Left	dp3		23.85					Juvenile	Subadult Juvenile			
K	AR 97 S42 93/Q44 71	Mand. Eq 1	Right	P3 P2							Juvenile	Subadult Juvenile			
K	AR 97 P56 1	Mand. Eq 2	Right	dp4 (P4 germ) M1			12.70				Juvenile	Subadult Juvenile			
K	AR97 K58 1/I42 2/N46 9	Mand. Eq 2	Left	dp4 (P4 germ)			13.30				Juvenile	Subadult Juvenile			
K	AR97 M53 13/R44 1	Mand. Eq 3	Left	M3							Prime Adult	Early Prime			
K	AR 96 I54 4	Mand. Eq 4	Right	M2							Juvenile	Subadult Juvenile			
K	AR 97 O45 1	Mand. Eq 5	Right	M2							Juvenile	Subadult Juvenile			
L	AR99 P42 1	Mand. Eq 1	Left	M3											
L	AR98 H58 7/AR97 N51 7/K56 4	Mand. Eq 2	Right	M1 M2 M3				42.73			Prime Adult	Early Prime	101.88	82.08	121.68
L	AR 97 K55 1/AR99 V48 465	Mand. Eq 3	Right	P2 P3											
L	AR99 S42 38	Mand. Eq 3	Left	P2											
M	AR00 S43 25/S43 18/AR02 N46 14/K54 16	Mand. Eq 1	Right	dp2 dp3 dp4 M3	13.99	9.68	14.98				Juvenile	Subadult Juvenile			
M	AR02 S50 55/S51 270	Mand. Eq 2	Right	M2 M3							Prime Adult	Early Prime			
M	AR02 L49 111/L54 19/L52 1/M51 2/L53	Mand. Eq 4	Left	P2 P3 P4 M1 M2 M3		53.57	56.21	43.20			Prime Adult	Early Prime	95.76	75	116.4
M	AR01/02 K48 9/L41 13/L47 1/K51 25	Mand. Eq 3	Left	P2 P3 P4 M1 M3	63.56			75.98		77.50	Prime Adult	Early Prime	63.6	51.84	75.24

**Table 7:** Number of equids maxillae at Abric Romaní, indicating level, archaeological reference, MNE, size, dental series, crown height of teeth, age group according to Stiner [5] and Bunn and Pickering [61], and mean, minimum and maximum age in month.

Level	Reference	Individual	Side	Dental series	Crown Height (mm)						Age group		Crown Height Method (Age months)		
					dp^2^/P^2^	dp^3^/P^3^	dp^4^/P^4^	M^1^	M^2^	M^3^	[5]	[61]	Mean Age	Minimum Age	Maximum Age
E	AR84 Q48 3/ S-T/48-49 11/AR98 O42 3	Max. Eq 1	Right	P3 P4 M3		45.45	48.92				Prime Adult	Early Prime	114.12	105	123.12
E	AR92 I64 24	Max. Eq 2	Left	M2					79.61		Juvenile	Subadult Juvenile	60	45.24	62.8
E	AR92 U48 275	Max. Eq 3	Left	Indeterminate											
H	AR90 CIII 2.2.5 L54 49/M54 28	Max. Eq 1	Right	M1 M2							Prime Adult	Early Prime			
H	AR90 CIII 2.2.5 L54 2/L54 4/L56 91/K58 1	Max. Eq 1	Left	P2 P3 P4 M1 M2			81.54				Juvenile	Subadult Juvenile	47.64	35.76	59.52
I	AR91 CIII J48 2/L47 2/N48 5	Max. Eq 1	Right	P2 P3 P4							Prime Adult	Early Prime			
I	AR91 N49 1	Max. Eq 1	Left	P2 P3 P4		72.08					Prime Adult	Early Prime	70.68	64.56	76.92
I	AR91 CIII H53 2/H52 1/H53 1/S53 3	Max. Eq 2	Left	P2 P3 P4 M2					85.09		Juvenile	Subadult Juvenile	38.52	25.68	37.32
I	AR91 R58 2/M49 2/J53 5	Max. Eq 3	Right	dp2 M1 M2	26.65			88.75			Juvenile	Subadult Juvenile	24	4.2	43.92
I	AR91 M58 1/S46 N/C	Max. Eq 4	Right	P2 P4							Prime Adult	Early Prime			
I	AR94 Niv I Q58 nº8	Max. Eq 5	Left	M1	94.73						Juvenile	Subadult Juvenile	27.96	18.6	37.32
Ja	AR93 N47 14/AR95 L51 140	Max. Eq 1	Left	P4 M1			27.95	21.05			Old	Old	222.72	212.16	233.28
Ja	AR93 R46 29/AR94 L48 1/AR94 N47 14/P48 26/R46 29	Max. Eq 2	Right	P2 P3 P4 M1	25.89	34.07		29.56			Prime Adult	Late Prime	144	132	156
Ja	AR94 P50 26/O47 117	Max. Eq 2	Left	P2 P4 P3 M1 M2	29.54	30.29			26.71		Prime Adult	Late Prime	156	144	168
Ja	AR95 I61 13/AR93 M55 2/AR93 M54 65	Max. Eq 3	Right	P2 P3 P4 M2 M3	38.69					66.98	Prime Adult	Early Prime	85.56	76.56	94.56
Ja	AR94 K55 8/AR94 F63 1/AR94 K37 107	Max. Eq 3	Left	P3 P4 M1 M2		58.48	67.35				Prime Adult	Early Prime	83.52	70.8	96.36
Ja	AR94 K54 17	Max. Eq 4	Left	P2	29.83						Prime Adult	Early Prime	133.92	120.84	147
Ja	AR94 K57 116/AR95 L49 142	Max. Eq 5	Left	dp2 M3	41.32					43.68	Prime Adult	Early Prime	113.16	100.44	126
Ja	AR98 N42 37/AR94 L57 141/AR93 N54 96	Max. Eq 6	Left	P2 P3 P4 M1 M2 M3	29.86	46.88	55.90		54.77	46.38	Prime Adult	Early Prime	110.64	99.24	121.92
Ja	AR93 K58 1/AR93 K56 1/AR96 J62 125	Max. Eq 7	Right	P2 P4 M1			53.34	60.75			Prime Adult	Early Prime	91.68	81.12	102.24
Ja	AR95 I62 15/AR94 K54 20/AR94 L57 211/AR98 N41 7	Max. Eq 7	Left	P2 P4 M1 M2 M3			50.53	60.77	48.53	43.42	Prime Adult	Early Prime	104.52	92.88	116.28
Ja	AR93 N54 nº97	Max. Eq 8	Right	P2 P3	33.61	42.90					Prime Adult	Early Prime	124.32	114.72	133.92
Ja	AR94 N48 25/AR94 K59 72/AR94 N54 154	Max. Eq 9	Right	P2 P3 P4 M1 M2 M3		58.49		61.25	65.84		Prime Adult	Early Prime	86.64	77.16	96
Ja	AR94 S53 19	Max. Eq 9	Left	P2							Prime Adult	Early Prime			
Jb	AR95 N53 2/M48 53/L55 4	Max. Eq 1	Right	M1 M2 M3						73.91	Prime Adult	Early Prime	71.76	59.04	84.48
Jb	AR95 N51 4/ M45 6/N51 58	Max. Eq 2	Left	P4M1M2M3			49.21	45.75	46.60		Prime Adult	Early Prime	113.88	95.76	132
Jb	AR95 M48 94	Max. Eq 3	Left	M3							Juvenile	Subadult Juvenile			
Jb	AR95 N49 48 /N51 8	Max. Eq 4	Right	M2 M3					50.71	52.53	Prime Adult	Early Prime	103.92	91.08	116.64
Jb	AR96 L48 6	Max. Eq 5	-	germ											
K	AR96 P52 8/N52 14/N52 13	Max. Eq 1	Right	P4 M1 M2			41.21	43.05	46.88		Prime Adult	Early Prime	123.72	112.44	135
K	AR97 N53 1/O52 56/N53 3/I52 15	Max. Eq 2	Left	P4 M1 M2 M3			37.85			49.22	Prime Adult	Early Prime	123.12	110.76	135.36
K	AR96 M45 1	Max. Eq 3	Right	M3							Juvenile	Subadult Juvenile			
L	AR97 J49 4/J49 9/O52 4	Max. Eq 1	Left	dp4 P4 M1			12.29	77.42			Prime Adult	Early Prime	61.92	52.56	71.28
M	AR02 K54 3/N54 125/K54 2	Max. Eq 1	Left	P4 M1 M2 M3							Prime Adult	Early Prime			
M	AR00/02 M51 1/J53 16/N49 9/ O47 2	Max. Eq 2	Right	P4 M1 M3						51.90	Prime Adult	Early Prime	98.76	85.92	111.48

**Fig 7:**
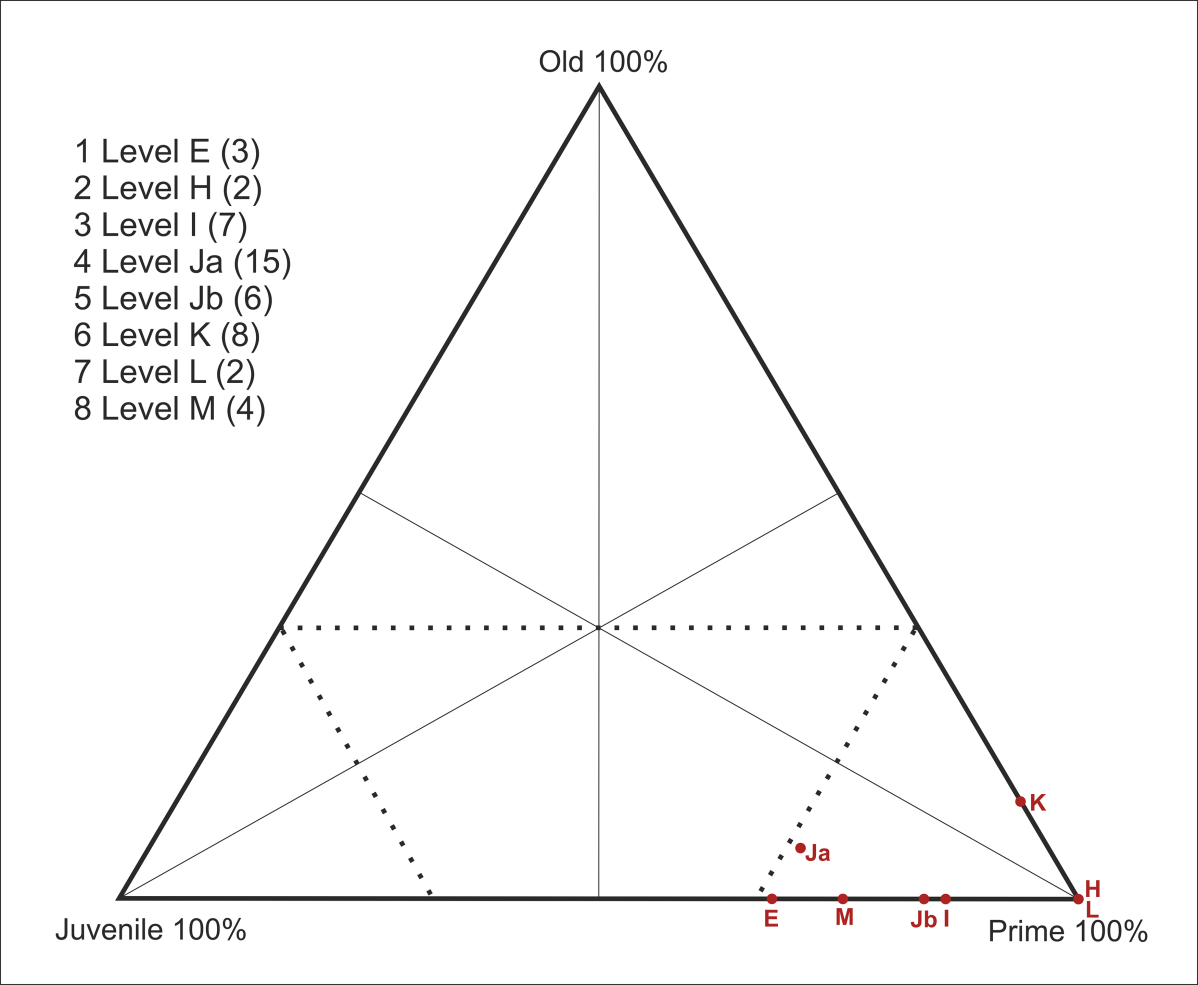
Triangular graph showing the age distribution of equid specimens by archaeological level (MNI).

A subadult juvenile individual, an early prime adult, and a late prime adult were identified in level E based on the determined age at death. Within level H, two early prime adult individuals were identified. Within level I, a subadult juvenile individual and six early prime adults were identified. Within level Ja, four subadult juvenile individuals, four early prime adults, six late prime adults and one old adult individual were identified. Within level Jb, a subadult juvenile individual, three early prime adults, and two late prime adults were identified. Within level K five early prime adults, two late prime adults and one old individual were identified. In level L two late prime adult individuals were identified. Within level M, a one subadult juvenile, two early prime adults and one late prime adult were identified. In total, 9 juveniles, 37 prime adults and 2 old adult individuals were identified (Table 5). The equids show a mortality profile that is dominated by prime adults within all levels of the sequence (Fig 7).

### Cervids

**Fig 8:**
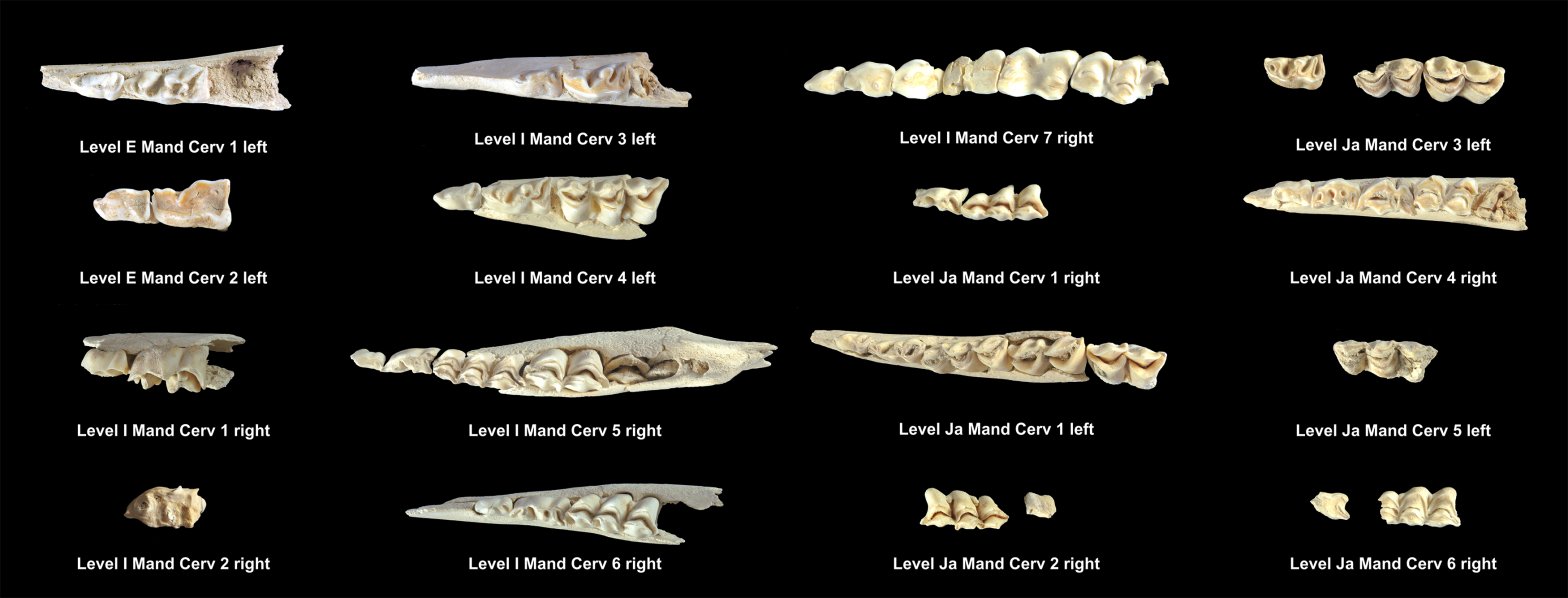
Cervid mandibles included in the MNI of level E, I and Ja. Under each dental series are references to level, individual and side as shown in Table 8.

**Fig 9:**
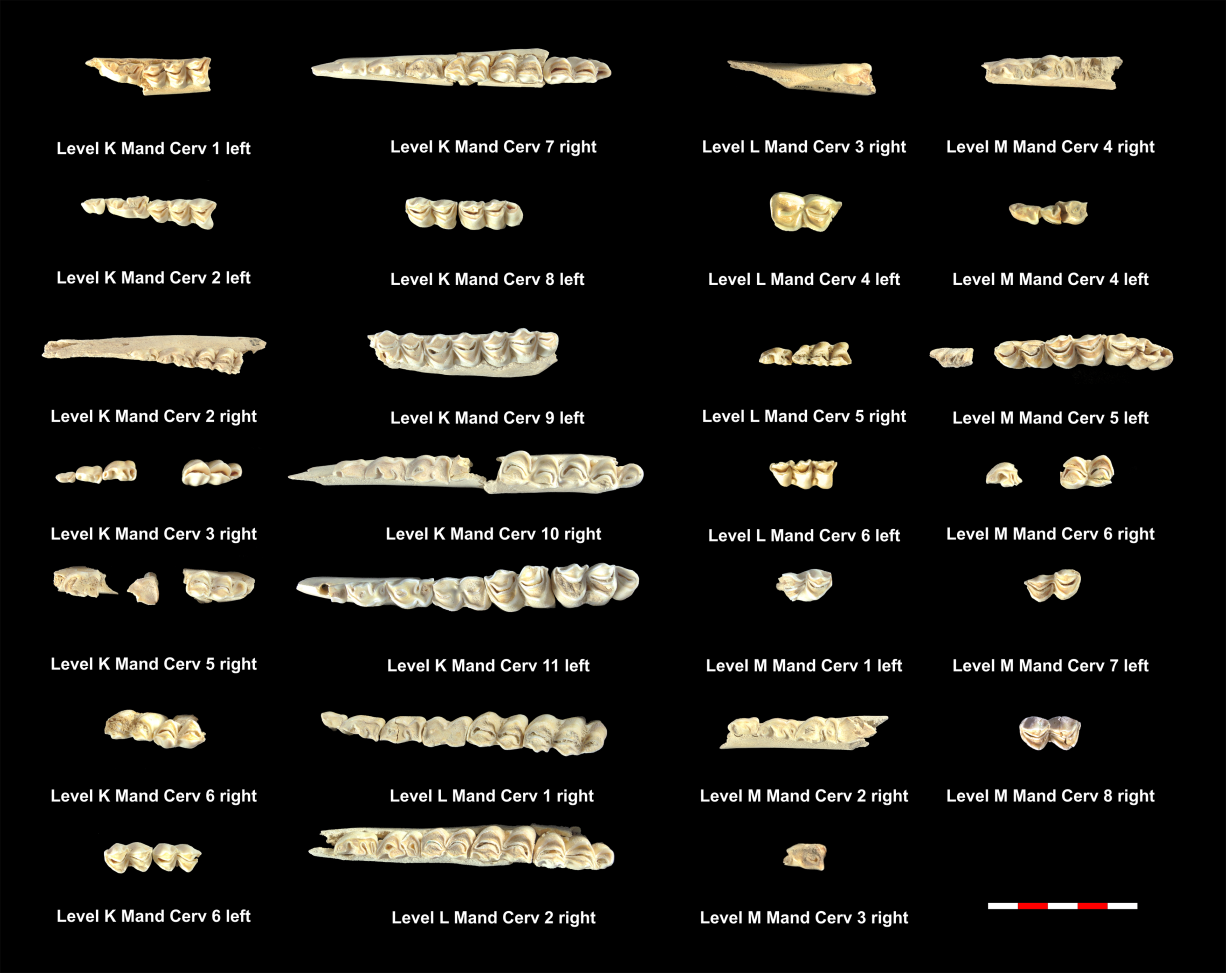
Cervid mandibles included in the MNI of level K, L and M. Under each dental series are references to level, individual and side as shown in Table 8.

**Fig 10:**
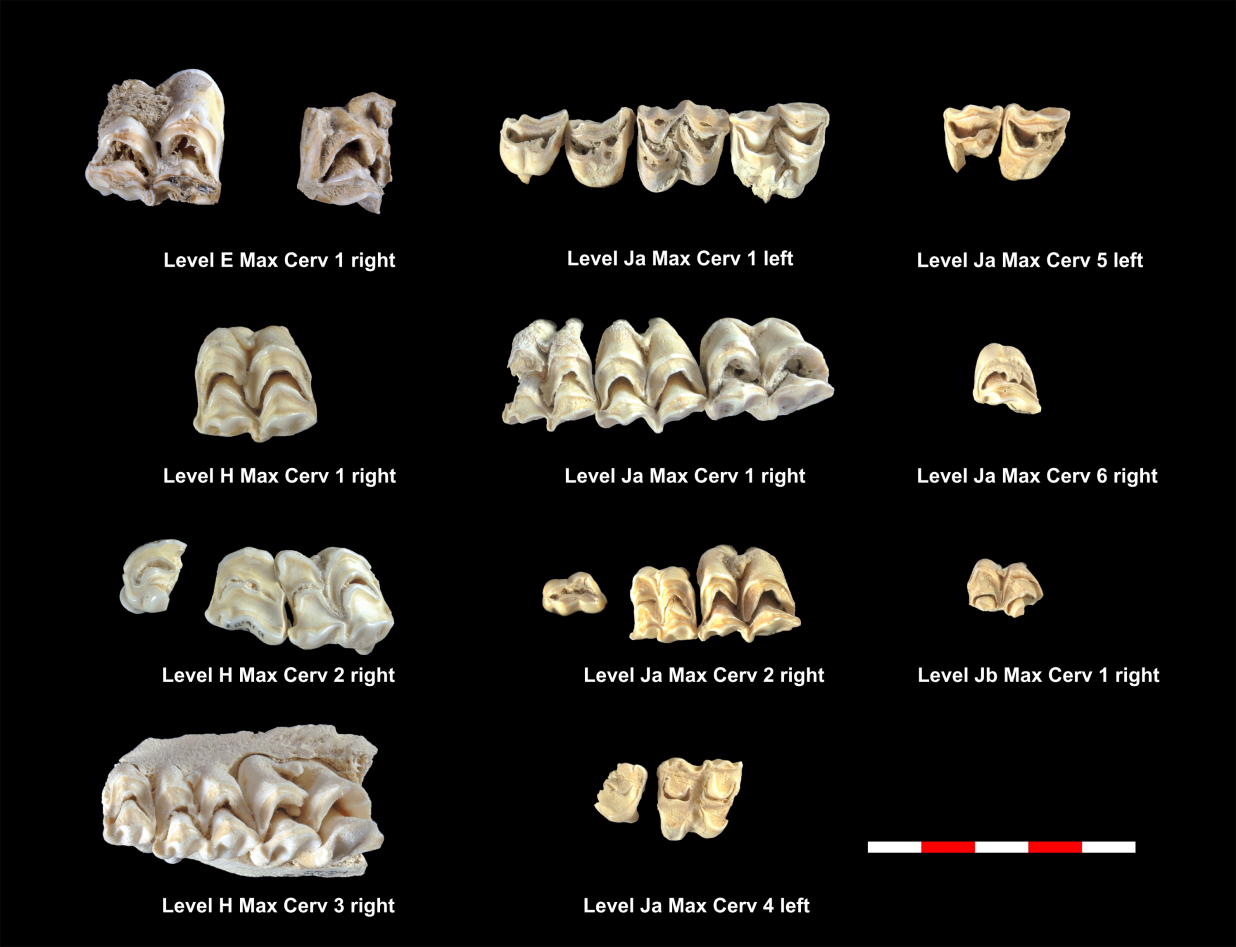
Cervid maxillae included in the MNI of level E, H, Ja and Jb. Under each dental series are references to level, individual and side as shown in Table 9.

Table 5 shows the total NISP, MNE and MNI calculated for the cervids found in each of the levels studied. Tables 8 and 9 show the MNE of mandibles and maxillae in each level. Figs 8–10 show the MNI of the cervids by level. Within each level, between one and 11 individuals have been identified (S1 Table). Level K and level M have the most individuals, whereas level Jb contains of the fewest. It was possible to define the wear pattern of the occlusal surfaces of 63 mandible teeth belonging to 34 individuals. The identified wear patterns are indicated in Tables 8 and 9.

**Table 8:** Number of cervid mandibles at Abric Romaní, indicating level, archaeological reference, MNE, size, dental series, crown height of teeth, code of wear stage, age group according to Stiner [5] and Bunn and Pickering [61] and mean age in month.

Level	Reference	Individual	Side	Dental series	Crown Height (mm)				Wear Stage				Age group		Quadratic Crown Height Method
					dp_4_	M_1_	M_2_	M_3_	dp4	M1	M2	M3	[5]	[61]	Age month
E	AR98 N41 209	Mand Cerv 1	Left	dp2 dp3									Juvenile	Young Juvenile	
E	AR92 T48 102	Mand Cerv 2	Left	P3 P2									Prime Adult	Early Prime	
I	AR92 L47 34	Mand Cerv 1	Right	dp2 dp3									Juvenile	Young Juvenile	
I	AR91 CIII I49 5	Mand Cerv 2	Right	dp3									Juvenile	Subadult Juvenile	
I	AR91 CIII J53 8	Mand Cerv 3	Left	P2 P3									Prime Adult	Early Prime	
I	AR92 K47 14/K47 15	Mand Cerv 4	Left	dp2 dp3 dp4	13.74				7L				Juvenile	Young Juvenile	0,0089
I	AR92 P inf. N.C.	Mand Cerv 5	Right	dp2dp3dp4M1 M2	7.56				12L	4A	0		Juvenile	Subadult Juvenile	5,50
I	AR92 L47 38	Mand Cerv 6	Right	dp2 dp3 dp4	13.03				4C				Juvenile	Young Juvenile	0,12
I	AR92 Plat. inf. BN	Mand Cerv 7	Right	P2 P3 P4 M1 M2	4.77					15A	13B	11G	Old Adult	Old Adult Class	165,58
Ja	AR92 L47 surface/AR92 Plat. Sup.	Mand Cerv 1	Right	dp2 dp3 dp4	10.42				7L				Juvenile	Young Juvenile	1,70
Ja	AR95 I63 17/AR98 L40 4	Mand Cerv 1	Left	dp2 dp3 dp4 M1 M2	12.43				7L	0	0		Juvenile	Young Juvenile	0,32
Ja	AR92 L47 surface/AR98 L40 3	Mand Cerv 2	Left	dp2 dp4	11.52				6L				Juvenile	Young Juvenile	0,81
Ja	AR94 P51 803/AR94 H51 1	Mand Cerv 3	Left	P3 M1 M2		16.42	23.75			6	3A		Juvenile	Subadult Juvenile	25,21
Ja	AR98 M42 12	Mand Cerv 4	Right	P2 P3 P4 M1 M2		10.70				9A			Prime Adult	Early Prime	63,58
Ja	AR92 Plat.Sup J	Mand Cerv 5	Left	dp4	8.61				6L				Juvenile	Young Juvenile	3,85
Ja	AR92 L47 surface	Mand Cerv 6	Right	dp3 dp4		6.44			11				Old adult	Late Prime	97,6
K	AR97 J47 24	Mand Cerv 1	Left	dp3 dp4	9.45				8				Juvenile	Young Juvenile	2,74
K	AR96 J44 4/J45 4	Mand Cerv 10	Right	P2 P3 P4 M1 M2 M3	9.98					14/15	9	11	Old adult	Old Adult Class	110,76
K	AR97 L49 26/K53 52	Mand Cerv 11	Left	P2 P3 P4 M1 M2 M3	9.21					9	8	9	Prime Adult	Early Prime	73,53
K	AR99 K40 s/c	Mand Cerv 2	Left	dp2 dp3 dp4				23.94	8				Juvenile	Young Juvenile	2,14
K	AR99 L43 4	Mand Cerv 2	Right	dp2 dp3 dp4					8				Juvenile	Young Juvenile	3,04
K	AR97 J45 17/J48 15/J46 9/J45 14	Mand Cerv 3	Right	dp2 P3 P4 M3				9.85				0	Prime Adult	Early Prime	40,21
K	AR96 U45 93	Mand Cerv 4	Right	M3			15.48	16.91				0	Juvenile	Subadult Juvenile	
K	AR99 H41 4 /AR97 J46 25/AR99 N42 10	Mand Cerv 5	Right	P3 M1 M3			14.88			15		11	Old Adult	Old Adult Class	122,16
K	AR99 N42 8	Mand Cerv 6	Left	M2 M3		15.77		19.61			9	6	Prime Adult	Early Prime	63,36
K	AR96 M47 34	Mand Cerv 6	Right	M2			19.59	22.25			9	6_7	Prime Adult	Early Prime	59,75
K	AR96 K54 s/c/N45 114	Mand Cerv 7	Right	P2 P3 P4 M1 M2 M3			13.53			8	5	8	Juvenile	Subadult Juvenile	44,96
K	AR96 N45 30	Mand Cerv 8	Left	M2 M3			8.64	10.73			5	5	Prime Adult	Early Prime	40,16
K	AR97 H45 1	Mand Cerv 9	Left	M2 M1 M3		8.33	13.93	16.50		9	8	9	Prime Adult	Early Prime	68,66
L	AR00 J42 nº8	Mand Cerv 1	Right	P2 P3 P4 M1 M2 M3		6.02	9.84	14.68		13	9	11	Prime Adult	Early Prime	94,29
L	AR97 O50 1	Mand Cerv 2	Right	P3 P4 M1 M2 M3		10.68	15.10			9	8	7	Prime Adult	Early Prime	61,05
L	AR 98 P47 4	Mand Cerv 3	Right	P2									Prime Adult	Early Prime	
L	AR99 T43 BN	Mand Cerv 4	Left	M3				4.38				12	Old Adult	Old Adult Class	175,26
L	AR00 J42 33	Mand Cerv 5	Right	dp3 dp4	10.46				1				Juvenile	Young Juvenile	1,66
L	AR98 I56 6	Mand Cerv 6	Right	dp4	11.60				1				Juvenile	Young Juvenile	0,76
L	AR99 K43 2	Mand Cerv 7	Right	M2			17.26				5		Prime Adult	Early Prime	45,90
M	AR01 T44 5 /R46 6/O45 4	Mand Cerv 1	Left	dp3 dp4	8.84				5 I				Juvenile	Young Juvenile	3,53
M	AR03 U51 638/640	Mand Cerv 2	Right	P2 P3 P4 M1		0				13			Old adult	Old Adult Class	164
M	AR00 R46 9	Mand Cerv 3	Right	P3									Prime Adult	Late Prime	
M	AR01 N46 NFC	Mand Cerv 4	Right	P3 P4 M1						I			Prime Adult	Late Prime	
M	AR01 R43 44/L48 42/ P43 4	Mand Cerv 4	Left	P2 P3									Prime Adult	Late Prime	
M	AR02 J55 1/AR00 N46 2/AR02 P53 53/K51 1	Mand Cerv 5	Left	P3 M1 M2 M3		15.58	20.31	22.33	7	5	5		Juvenile	Subadult Juvenile	37,09
M	AR02 U46 12, 13/K50 7	Mand Cerv 6	Right	P3 M1		11.10				9			Prime Adult	Early Prime	60,79
M	AR01 Q45 30	Mand Cerv 7	Left	M1						5			Prime Adult	Early Prime	
M	AR02 J52 43	Mand Cerv 8	Right	M1		19.50				4			Juvenile	Subadult Juvenile	18,19

**Table 9:** Number of cervid maxillae at Abric Romaní, indicating level, archaeological reference, MNE, size, dental series, crown height of teeth, code of wear stage, age group according to Stiner [5] and Bunn and Pickering [61] and mean age in months.

Level	Reference	Individual	Side	Dental series	Crown Height (mm)				Wear Stage				Age group		Quadratic Crown Height Method
					dp^4^	M^1^	M^2^	M^3^	dp^4^	M^1^	M^2^	M^3^	[5]	[61]	Age month
E	AR92 T48/CIII/AR98 N40 16/N41 123	Max Cerv 1	Right	M1 M2		20.3				4A			Prime Adult	Early Prime	6.88
H	AR91 2.2.5 L59 3	Max Cerv 1	Right	M3				13.12				8A	Prime Adult	Late Prime	
H	AR91 2.2.5 L59 /L59 5	Max Cerv 2	Right	P3 P4 M1		7.8				8			Prime Adult	Late Prime	82.71
H	AR84 M45 1	Max Cerv 3	Right	M1 M2 M3			8.84				5A	3B	Prime Adult	Late Prime	67.59
I	AR91 Pinf I58/ S53 BN	Max Cerv 1	Left	P2 P3 P4 M1M2		4.37				9A			Old adult	Late Prime	124.9
I	AR92 L46 5 / M45 4 / L46 7	Max Cerv 2	Left	dp2 dp3 dp4 (P2 P3 P4) M1 M2 M3	6.62	15.67		23.27		4A	2A		Juvenile	Subadult Juvenile	20.7
I	AR92 K46 31 / K46 30	Max Cerv 3	Right	dp2 dp3									Juvenile	Subadult Juvenile	
I	AR92 M46 4	Max Cerv 4	Left	dp2 dp3 dp4	12.92				4A				Juvenile	Young Juvenile	0.27
Ja	AR95 I49 6/I49 7/AR93 H46 12	Max Cerv 1	Right	M1M2M3		8.51	15.05	21.77		5	4A	2A	Prime Adult	Early Prime	75.12
Ja	AR93 K57 34/K57 35/L43 10/L50 352	Max Cerv 1	Left	P2 P3 M1 M2						5A			Prime Adult	Early Prime	
Ja	AR94 L50 2/AR93 M46 8/AR93 M46 6	Max Cerv 2	Right	dp2 dp4 M1	6.79	15.62			14L	4A			Juvenile	Subadult Juvenile	20.94
Ja	AR94 N53 302	Max Cerv 3	Right	dp2									Juvenile	Subadult Juvenile	
Ja	AR95 I62 65/AR94 K59 106	Max Cerv 4	Right	M1P4		9.73				4A			Prime Adult	Early Prime	63.01
Ja	AR95 I49 8/AR93 N55 30	Max Cerv 5	Right	P2 P3									Prime Adult	Early Prime	
Ja	AR95 I49 8	Max Cerv 6	Right	P3									Prime Adult	Early Prime	
Ja	AR92 Plat. Sup./AR 94 M49 12	Max Cerv 7	Left	dp2 dp3 dp4	14.56				4A				Juvenile	Young Juvenile	0.003
Ja	AR94 L48 13	Max Cerv 8	Right	dp3 dp4 M1	10.84	18.48			7	2A			Juvenile	Young Juvenile	10.31
Jb	AR93 N51 76	Max Cerv 1	Right	M1		8.11							Prime Adult	Late Prime	79.35
K	AR96 M45 71	Max Cerv 1	Right	P2 P3 P4 M1 M2 M3		11.03	14.68	18.83		6	4	4	Prime Adult	Early Prime	51.39
K	AR96 J47 21	Max Cerv 1	Left	M1 M3 M2		10.91	17.06	20.75		6	4	4	Prime Adult	Early Prime	52.41
K	AR96 N46 20/J49 1/J54 2	Max Cerv 2	Right	P2 M1 M2			13.03						Prime Adult	Early Prime	33.13
K	AR96 M52 5/K49 4	Max Cerv 3	Left	dp4 M1	5.22	16.63			14L	5			Juvenile	Subadult Juvenile	16.46
K	AR99 I42 4	Max Cerv 4	Left	P4									Prime Adult	Early Prime	
L	AR00 K42 3/AR97 S50 2	Max Cerv 1	Left	P2 P3 M1		9.25							Prime Adult	Early Prime	67.64
M	AR00 S41 5	Max Cerv 1	Right	M1 M3 M2			11.3	18.08		5	5	1	Prime Adult	Early Prime	45.67
M	AR02 T44 47/T44 51	Max Cerv 2	Left	P2 P3 P4 M1 M2 M3		8.38	16.48	18.42				4	Prime Adult	Early Prime	76.48
M	AR00 P43 30/ M52 2	Max Cerv 3	Left	P2 P3 P4 M1 M2 M3		9.32		18.71		9	6	4	Prime Adult	Early Prime	66.95
M	AR00 S43 23	Max Cerv 4	Right	P4 M1M2		17.72	20.92			4	2		Juvenile	Subadult Juvenile	12.51
M	AR01 M41 48/K50 20/AR02 N48 96/AR03 U49	Max Cerv 4	Left	P3 P4 M1 M2						4	2		Juvenile	Subadult Juvenile	
M	AR01 M44 12/AR03 U50 453	Max Cerv 5	Left	P3 P4									Prime Adult	Early Prime	
M	AR01 O43 109/N45 20	Max Cerv 6	Right	P2 P3									Prime Adult	Early Prime	
M	AR02 K54 25/AR01 N45 23/N46 101	Max Cerv 7	Right	P2 P3 P4 M2			6.77				9A		Prime Adult	Late Prime	89.76
M	AR00 N47 41	Max Cerv 7	Left	M2									Prime Adult	Late Prime	

Based on the determined ages at death a subadult juvenile individual and two early prime adults were identified within level E. Within level H, three late prime adults were identified. Within Level I, four young juveniles, a subadult juvenile, an early prime adult and an old adult individual were identified. Within level Ja, three young juveniles, two subadult juveniles, four early prime adults and one late prime adult were identified. Within level Jb, only one early prime adult has been identified. Within level K, two young juveniles, two subadult juveniles, four early prime adults, one late prime adult and two old adult individuals were identified. Within level L, two young juveniles, three early prime adults, one late prime adult and one old adult individual were identified. Within level M, a young juvenile, two subadult juveniles, two early prime adults, two late prime adults, and one old adult individual were identified. In total, 20 juveniles, 25 prime adults and 5 old adult individuals were identified (Table 5).

**Fig 11:**
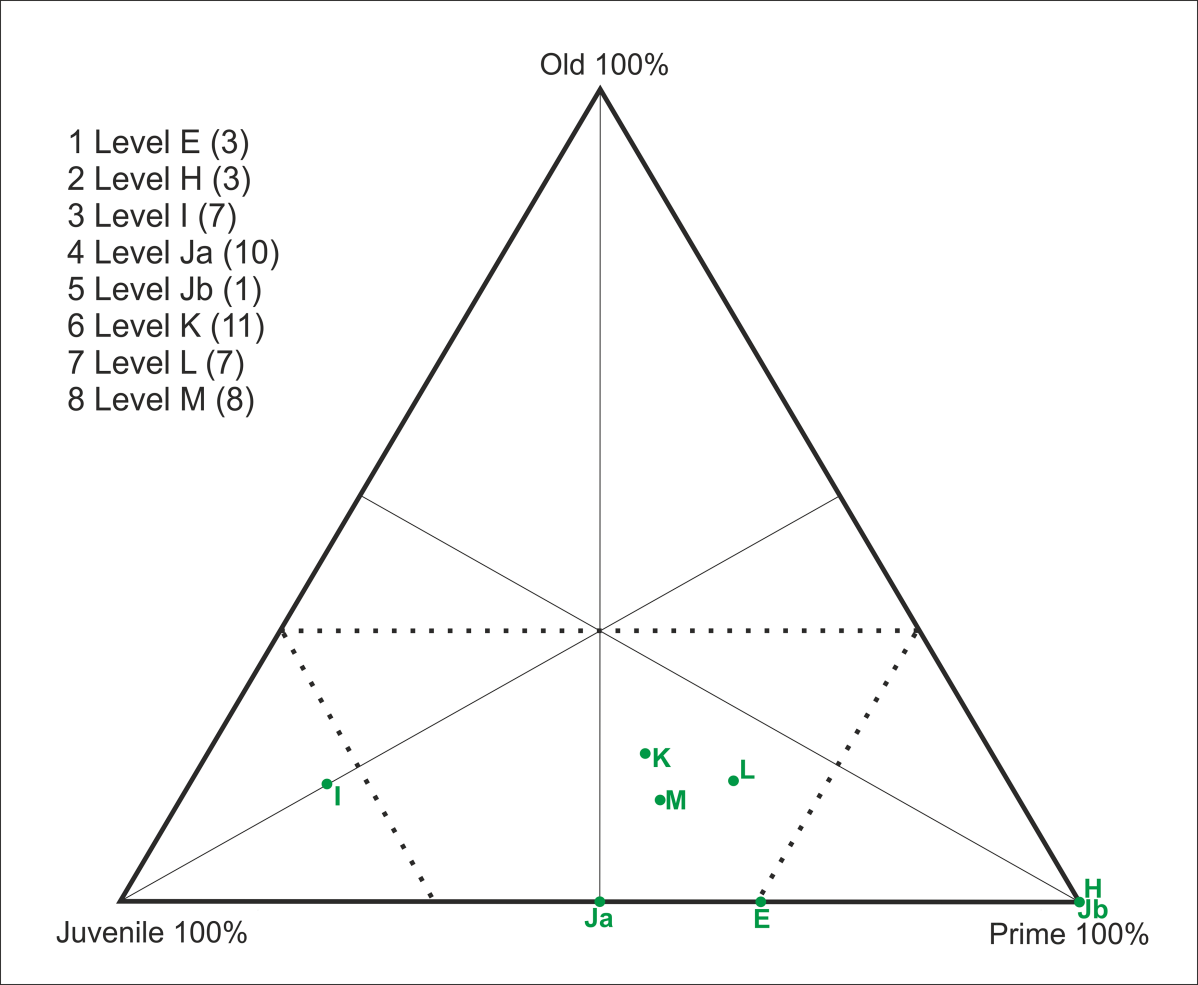
Triangular graph with the age distribution of cervid specimens according to archaeological level (MNI).

For the cervids, the mortality profiles vary by level, and five different models have been identified (Fig 11): 1) a profile that is located at the intersection between the catastrophic mortality and the prime-dominated profiles in level E; 2) a profile that is dominated by prime adults within levels H and Jb; 3) a mortality profile that is dominated by juveniles in level I; 4) a profile that is positioned at the intersection between the attritional and catastrophic profiles at level Ja; 5) and a catastrophic mortality profile obtained from levels K, L and M (Fig 11).

## Discussion

**Fig 12:**
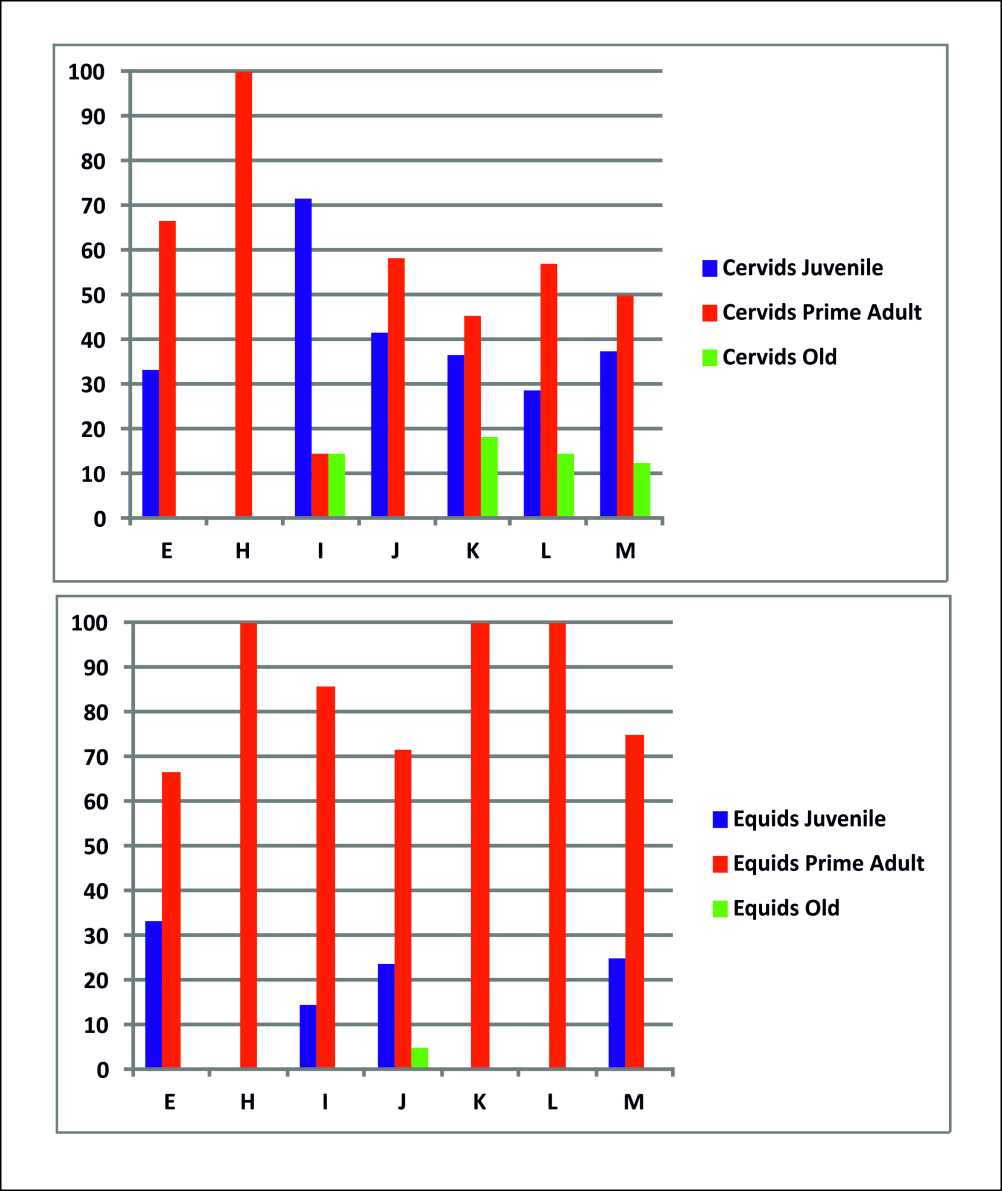
Percentage of total age group of equids and cervids by archaeological level.

To date, the deposits uncovered at Abric Romaní have yielded a sequence of 15 levels formed during the interstadial MIS 3. An exclusively anthropogenic origin for the lithic and faunal remains has been inferred for all of these levels. The mortality profiles of the main taxa indicate the existence of marked differences between the ages of the equids and cervids. The equid accumulation is dominated by prime adults, whereas the cervid accumulation presents greater variability among the different levels (Figs 7, 11 and 12).

None of the mortality profiles identified at Abric Romaní are unusual in the context of the European Middle Paleolithic record [5, 28, 37, 43, 53, 54, 116, 117]. Mortality profiles that differ according to the taxa hunted have been found at the Manie, Madonna and Lazaret sites [53]. At Manie and Madonna, aurochs present prime-dominated profiles, whereas red deer present a catastrophic profile; however, that difference only exists in one level at each site. At Lazaret, the profiles of deer and ibex change in each of the site’s five levels, and neither species has a regular profile [53]. At Abric Romaní, two mortality profiles have been observed throughout the studied sequence. In addition, the atrophic character of the accumulation suggests that the mortality profiles could be considered a valid proxy that can be used to infer the hunting strategies used by the Neanderthals of Abric Romaní.

However, inferences made using mortality profiles should be taken with caution as they may not accurately reflect prey selections made by hominins [118]. Bone accumulations are affected by two key processes, specifically the possible enhanced destruction of young individual teeth and the differential transport of heads according to the weight of individual prey animals. These processes may affect the representation of juvenile individuals in a few different ways: a) because their teeth tend to disappear easily; and b) because their lower weight means that they are more likely to have been transported whole to the site [118].

Considering the differential preservation of individual as a function of age, the scarcity of juvenile equids (17% of the sample) seems to be due to differential destruction processes. However, the high abundance of juvenile cervids (40% of the sample), whose teeth have a lower density than those of equids, indicates that the bias toward adult horses is likely not the product of differential preservation processes, because it should also have altered the deer sample.

Regarding the possible differential transport of heads, Marean [118] indicated that three interrelated factors should be considered: 1) the weight of the animal; 2) the size of the hominin group; 3) the distance between the kill site and the reference site.

**Fig 13:**
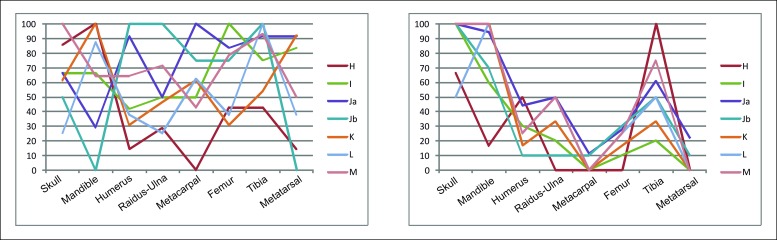
Anatomical profiles of the cervids (left) and equids (right) from the levels of Abric Romaní.

Depending on the weight of the animal, many ethnoarchaeological studies indicate that hunter-gatherers faced with equal conditions usually transport more complete small animals than large animals [119–121]. In addition, the variation in the weight of an animal species throughout its life must also be considered, as it affects the decision to transport the heads of that species or not, and therefore also the composition of the mortality profiles [118]. In equids, we documented a predominance of large adult individuals (MNI = 39) over medium-sized young individuals (MNI = 8) (Table 5), while among the cervids we found no great differences between the representation of small (MNI = 20) and medium-sized individuals (MNI = 30) (Table 5). Also, the anatomical representation indicates that the cranial elements of the large animals were transported in equal proportion, or even more, to the rock shelter than cranial elements of medium-sized animals (Table 10) (Fig 13). In levels Ja, Jb, K and M the value of %MAU for skulls is 100% and in levels H and L it is equal or superior to 50%. However, for the cervids the value is 100% only in level M, and in level L the value is even under 30% (Fig 13). MNE and %MAU data for level E are not available. Thus, the weight of the animals did not appear to be a major determining factor in the transport of their heads. However, is important to consider that this circumstance could have affected the transport events [122], because the superposition of diverse transportation strategies has been identified at Abric Romaní, due to multiple conditioning factors, such as the sizes of different animals and the food utility of the transported elements [94].

**Table 10:** MNE of high survival elements of the cervids and equids from the levels of Abric Romaní [73, 82, 88, 90, 94].

	E		H		I		Ja		Jb		K		L		M	
	Equids	Cervids	Equids	Cervids	Equids	Cervids	Equids	Cervids	Equids	Cervids	Equids	Cervids	Equids	Cervids	Equids	Cervids
Skull	3	1	2	3	5	4	9	8	5	1	3	4	1	1	2	7
Mandible	2	2	1	7	6	8	17	7	7	0	6	13	4	7	4	9
Humerus	-	-	3	1	3	5	8	22	1	4	1	4	1	3	1	9
Radius-Ulna	-	-	0	2	2	6	9	12	1	4	2	6	2	2	2	10
Metacarpal	-	-	0	0	0	6	2	24	1	3	0	8	0	5	0	6
Femur	-	-	0	3	1	12	5	20	3	3	1	4	1	3	1	11
Tibia	-	-	6	3	2	9	11	22	5	4	2	7	2	8	3	13
Metatarsal	-	-	0	1	0	10	4	22	1	0	0	12	0	3	0	7

An ethnoarchaeological principle used in the study of mortality profiles indicates that, under equal conditions, within an average settling system of small groups, hunter-gatherers show greater selectivity in making transport decisions, and often abandon the heads of large animals [119, 120, 123]. On the other hand, in an average settlement system of large groups, the bias of the transport of these animals may be reduced [118–121]. The spatial patterns documented within the Abric Romaní correspond to bivouacs around isolated external hearths and to camps with many external hearths connected by reassembling, and resting and sleeping areas [78, 92, 124]. All the levels are the result of an unknown number of occupation events that occurring over hundreds to thousands of years. The levels originating from short-term or non-residential occupation events have been defined as the result of the activity of groups of hunter-gatherers immersed within a regional foraging mobility model, whereas the long-term events have been interpreted as a result of the activity of medium or large groups [70, 81, 82, 84, 91]. Levels H, I, J, K, L, and N have been defined as short-term occupation events associated with a highly mobile Neanderthal group. Analysis of the distribution and nature of the combustion structures associated with the sleeping and resting areas of level N, suggest that it could correspond to a Neanderthal group of between 8 and 12 individuals. Monahan [125] indicates that the Hadza require at least 10 to 12 porters to transport size 3 animals (113–340 kg) or greater. At Abric Romaní, the occasional transport of complete carcasses of medium-sized and large animals has been identified in all of the levels, regardless of the occupation model. This indicates that hunting parties may have been sufficiently numerous to transport a complete or almost complete large animal [94], at least occasionally.

In keeping with that suggested by Marean [118], differences in the transport of heads may be present within the sequence of Abric Romaní, depending on whether small or large groups occupied the rock shelter.

Level I is the result of short-term or non-residential occupations events, in which the mobility of the group was directly related to the exploitation of hunting resources [91]. The mortality profile of cervids shows a greater number of young individuals (MNI = 5) than adults or old individuals (MNI = 1/1). The predominance of young animals may be due to selective transport, as suggested by Marean [118], in which the transport of small individuals takes precedence over the transport of the heads of adult deer. However, at this level, we see that the transport of equids is not governed by this principle. The equids are represented by one young individual and six prime adult individuals; thus, the large animals are more abundant than medium-sized animals. Therefore, the age representation of both species does not appear to be due to the selective transport of heads, according to the weight of the animal.

On the other hand, level J has been identified as resulting from of long-term occupation by medium or large groups composed of more than 12 individuals, and may be related to the aggregation of groups of hunter-gatherers [93]. We found a balance in the representation of small and medium-sized animals among the cervids in this level. Meanwhile, the equids are dominated by large prime adults, which is common to all the levels. Thus, in spite of differences in the settlement model, age selection does not seem to be conditioned by animal size.

Finally, another factor that would condition the transport of the heads is the distance between the kill/butchering site and the reference site. The Abric Romaní is located inside an ecotone that is formed by the Anoia River, the plains that surround it, and the mountains that delimit the gorge. These features give the site strategic importance, and from it, the Neanderthals had immediate access to different biotopes and species [68, 86, 93, 126]. Biotic resources, both faunal and vegetation-based, were locally exploited [86, 89]. The selection of prey mainly focused on obtaining two types of animals, cervids and equids, which may indicate that the axis of mobility was oriented toward the open areas and the forests on the mountainsides that enclose the river valley [68, 86, 89, 93, 126]. In any case, assuming long distances from the kill/butchering site to the reference site, would favor a bias toward large animals. However, as mentioned, the small and medium-sized cervids are represented approximately equally, whereas there is a dominance of large animals among the equids. Therefore, either the transport distance associated with the equids was shorter, or the transport of their heads did not constitute a problem.

Although the archaeological mortality profile does not necessarily correspond to the original mortality profile [118], it seems that the mortality profiles documented at Abric Romaní closely reflect the prey selection and hunting strategies and behaviors of the Neanderthals who occupied the shelter.

**Fig 14:**
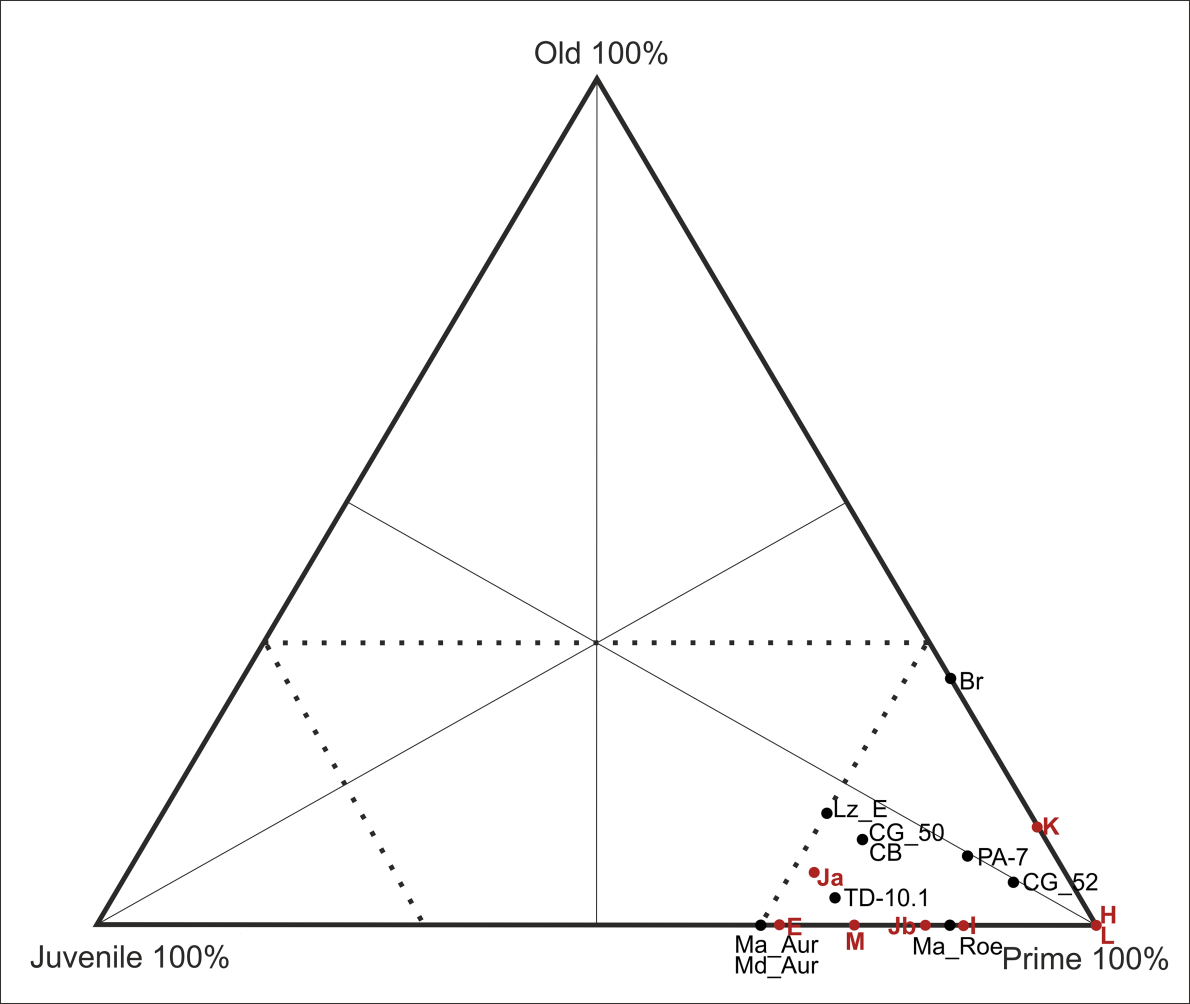
Triangular graph of mortality profiles of equids from different levels at Abric Romaní (E, H, I, Ja, Jb, K, L and M) and from different Early and Middle Paleolithic sites. Lz E = Lazaret [53]; Md_Aur = Madonna aurochs [53]; Ma_Aur, Ma_Roe = Manie aurochs and roe deer, respectively [53]; Br = Breuil [53]; CG 50, 52 = Combe Grenal [52]; PA-7 = Pech-de-l’Aze I [54]; CB = Cuesta de la Bajada [60].

Prime-dominated profiles like those observed at Abric Romani have also been identified for different species and at numerous archaeological sites, such as equids at Cuesta de la Bajada, aurochs at Manie and Madonna, and cervids at Combe-Grenal, Lazaret E, Pech-de-l’Aze I level 7, Breuil and TD10.1 (Fig 14) [5, 27, 28, 52–54, 59, 60].

Considering the predominance of a particular age group among the equids, we argue that individual animals may have been selectively hunted. However, Bunn and Gurtov [127] have indicated that, when a group of hunter-gatherers has sufficiently advanced technology to hunt individuals of any age group, prime adults will be captured more frequently because they are the most numerous individuals in prey populations, and not because the hunters are selective. If true, this principle should also govern the age selection of cervids. However, we observed that this premise is not fulfilled (Fig 6). Other studies have argued that the preference for prime adults reflects the objective of maximizing the return rates of predation events [63, 128]. Thus, we assume that the ability to take game from any age group would result in a preference for larger, more profitable prime adults, rather than young animals [63]. The presence of a stable predation model for equids and a variable model for cervids throughout the sequence seems to be related to the intentional selection of prime adult equids.

The identification of primaries access, the immediate and recurrent access to large carcasses (over 300 kg), has been interpreted as the result of cooperative hunting [27, 28, 58, 59, 125]. In addition, this group cooperation has already been demonstrated at Abric Romaní through an analysis of the transport strategies used for animal carcasses [94]. The mortality profiles of the equids, which are dominated by large animals, support cooperative and selective hunting as the social organization of the hunting groups.

Studies of the micro-wear of the teeth of equids from Abric Romaní indicate that they were hunted for short periods of time within the same season, although not synchronously [86, 90]. Thus, we can reject the hypotheses of mass and multiple predations, and assume that simple predation events were most commonly carried out by the Neanderthal groups.

Several types of hunting techniques have been proposed that produce a prime-dominated profile. Prime-dominated profiles have been associated with selective ambush hunting in different ethnoarchaeological and archaeological studies where primary access to animal carcasses has been documented [1, 5, 24, 28, 61, 129]. Bunn and Pickering [129] have proposed ambush hunting for the FLK Zinj assemblage (Bed I of Olduvai), where the mortality profile of the great ungulates is dominated by prime adults.

Binford [24] observed that the Nunamiut generated prime-dominated profiles of caribou on certain occasions. They established hunting camps in narrow passages along the caribou migration routes, and captured the animals using bows and arrows or rifles. Thus, the hunting of prime adults was made possible by the planned use of space (i.e. the interception of prey in specific places) and by cooperative work during the acquisition of carcasses [24]. Therefore, we can argue that the hunting of horses by the Neanderthals of the Abric Romaní was accomplished by selective and cooperative hunting tactics, possibly by ambush hunting, although this conjecture cannot be demonstrated empirically.

**Fig 15:**
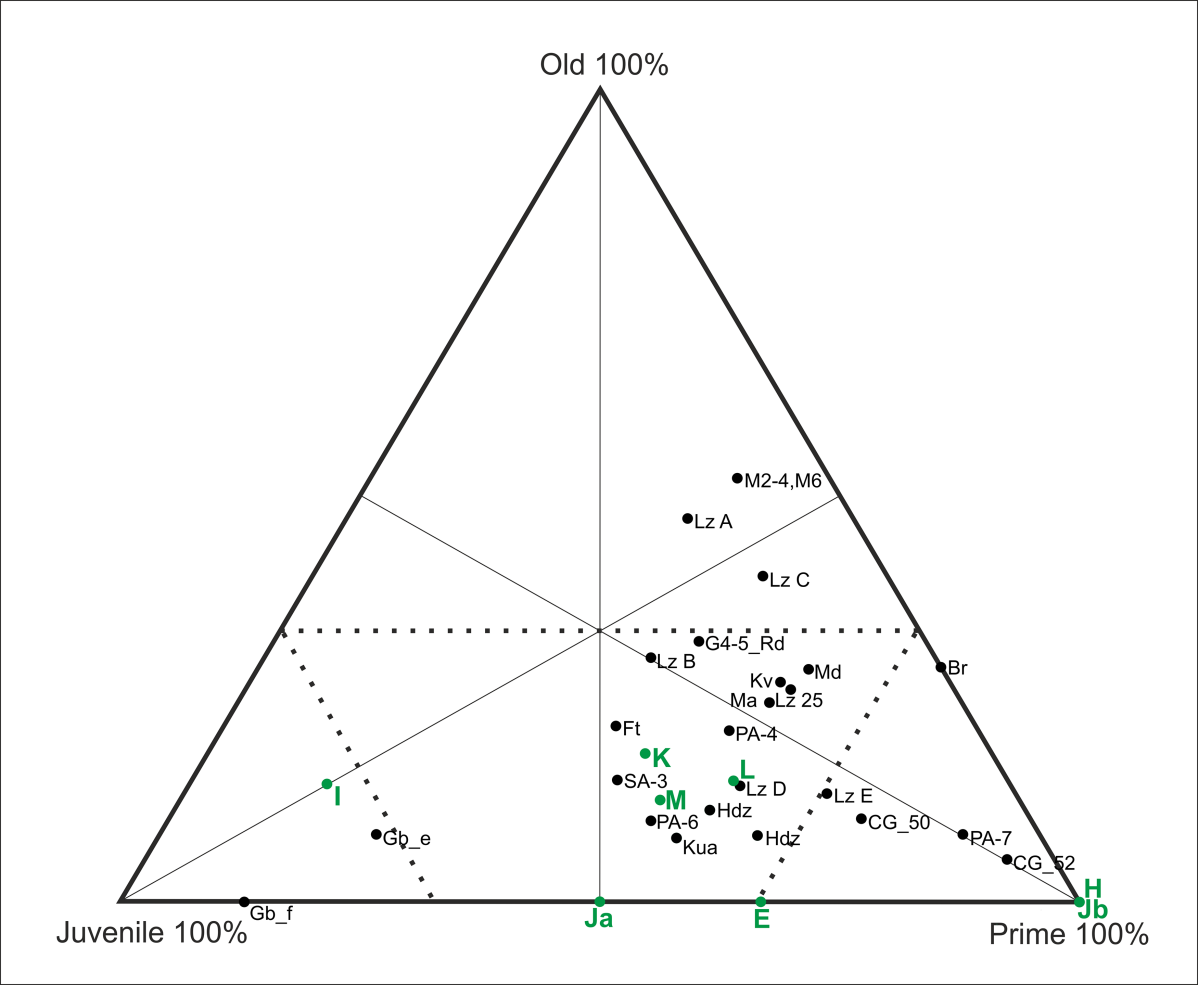
Triangular graph of mortality profiles of cervids in different levels at Abric Romaní (E, H, I, Ja, Jb, K, L and M) and cervids at different Middle Paleolithic sites. Gb e/f = Gabasa levels [52]; Ft_Rd = Fate red deer [27]; Lz A, B, C, D, E, 25 = Lazaret levels [53]; Md = Madonna [53]; M2-4, M6 = Moscerini levels [53]; Kv = Kevara [27]; Ma = Manie [53]; Br = Breuil [53]; CG 50, 52 = Combe Grenal levels [52]; PA 4, 6, 7 = Pech-de-l’Aze levels [54]; Hdz = Hadza assemblages; K = Kua [127].

The mortality profile of the cervids is characterized by great internal variability. Although it is widely assumed that Neanderthal activities generated prime dominated profiles, the mortality profiles of the cervids in the Abric Romaní sequence are not unusual within the Middle Paleolithic record (Fig 15). The mortality profile of the cervids shows both selective (levels E, H, I and Jb) and non-selective (levels Ja, K, L and M) patterns.

As indicated by Stiner [27], the mortality patterns generated by the hunting activities of human groups usually range from non-selective with an average towards the prime adults to heavily biased toward the prime adults. These patterns appear to be reflected in the cervid profile of level E, which is located between the intersection of the catastrophic and prime-dominated profiles (Fig 15). A prime-dominated profile has been identified at levels H and Jb (Fig 15). Although the cervids are only represented by three and one individual in these levels, respectively, which makes it difficult to defend a clear tendency in the selection of prey, prime-dominated profiles point to an age-selective hunting strategy [5].

Level I yielded a mortality profile that is dominated by young individuals (Fig 14). In this scenario, the Neanderthals would have deliberately hunted younger animals, which have the lowest economic return rates [130]. The preference for the young individuals of *Cervus elaphus* is also found in other Mousterian sites, such as Cova Beneito levels XII-X (Spain) [131] and Gabasa (Spain) [52]. In the latter case, once an increase in the presence of juvenile individuals by the action of other carnivores was ruled out, Steele [52] suggested that the greater presence of juvenile individuals is related to Neanderthal activity. The same behavior could account for the accumulation of cervids in level I, which includes four individuals with PEL values of less than 2.7% (less than 5 months of age). Measurement of the crown heights of these individuals indicates that two had not even passed the first month of age (Tables 8 and 9). This particular accumulation suggests a specialized seasonal hunt, perhaps during the same occupational event, indicating that hunting events were more common during cervids birthing season. Varin [132] has indicated that due to the presence of newborns, female groups display a pattern of low mobility with very specific habits in which they follow established paths at precise times of day. This behavior makes these animals extremely easy to track [54], so hunters may have waited for female groups to take their regular route and hunted them using ambushes or traps. The hunting of young individuals has been documented among current groups of hunter-gatherers. During the dick-dick and steenbock birthing seasons the! Kung San of the Kalahari capture young individuals by chasing them on foot or throwing clubs [133].

Binford [24] observed hunting events in which the Nunamiut (Tulekana and Kakinya) exclusively hunt young reindeer in order to obtain soft leather for clothing. Lithic use-wear analyses at Abric Romaní show that worked skins existed within the sequence, with work on fresh leather being more common [134]. In addition, lithic functionality studies in level Ja relate denticulate and notch features to the hardening of hides [81]. In the Abric Romaní sequence, although young individuals have been identified in almost all of the studied levels, they do not reach 71% of the total, as in level I. Therefore, in this level, the hunting of cervids seems to have been specifically intended to obtain this prey of low economic return, possibly to obtain their hides.

The mortality profile of level Ja is located at the intersection between the attritional and catastrophic profiles, indicating equal proportions of juvenile and prime adults, but no old adult individuals (Fig 15). The occupations took place in different events throughout the autumn and early winter, generally during a whole season, as indicated by studies of the seasonality of the ungulates [93]. Thus, the mortality profile ranged from attritional to catastrophic, indicating that Neanderthals captured individuals ranging from the weakest to the strongest over long periods of time, thus reflecting non-selective hunting.

Catastrophic mortality profiles have been identified at levels K, L and M (Fig 15). Catastrophic mortality profiles have also been observed for *Cervus elaphus* at the Mousterian sites of Pech-de-l'Aze (France) in levels 4 and 6 [54], Manie (Italy), Madonna (Italy), Fate (Italy), Lazaret (France) [53], Kebara (Israel), Sant'Agostino 3 and Guattari G4-5 (Italy) [5, 27]. Traditionally, catastrophic mortality profiles have been considered to illustrate mass communal hunting events or repeated episodes of non-selective hunting [2, 3, 5, 25, 127]. The faunal assemblages produced by mass communal hunting events are characterized by large accumulations made up of dozens of individuals of the same species, almost monospecific [25]. The systematic transport of high utility elements to the base camps has been documented in these assemblages [25]. The characteristic mortality profile of these sites is catastrophic, as determined by the age and sex of the individuals represented, with a strong trend toward seasonal or synchronous death [25]. In levels K, L and M more than three taxa are present (Table 1) with between 1 and 11 individuals of each species. The transport of the anatomical elements reveals a close correlation between the anatomical elements present and their richness in unsaturated marrow [94]. The cervids found in levels K and L were hunted over an estimated period of three months; those found in level M appear to have been hunted over an estimated period of about six months [86]. These analyses indicate that the cervids found in levels K, L and M were not hunted synchronously. Therefore, the catastrophic mortality profile of the cervids found in levels K, L, and M did not result from mass communal hunting events. As an alternative explanation for catastrophic profiles, Rendu [54] proposed that the lack of clear seasonality may be the result of a palimpsest, in which different seasonal events accumulate and finally generate a catastrophic mortality profile. However, if this explanation were to apply in the case of Abric Romaní, the equids should have catastrophic profiles as well as the cervids, since the sets are the result of multiple occupational events with a broad seasonal distribution that can occupy an entire season, as in levels Ja, K, L or M [86, 91, 93]. Thus, the differences between the cervids and equids in levels Ja, K, L and M tend to support the hypothesis of the non-selective hunting of cervids, suggesting that different tactics were used to hunt different taxa and were maintained over time, and they did not result from equifinal processes.

Thus, the mortality profile generated by the Neanderthals for the cervids indicates a less selective hunting strategy regarding the age of this taxon, and is more closely related to its abundance in the environment and therefore to the rates at which hunting groups would have encountered this animal [135, 136]. In terms of the organization of hunting groups [65], once the possibility of communal hunting has been dismissed, it is impossible to determine whether the capture of the animals was accomplished by cooperative hunting parties or individuals. However, because cooperation has been established as necessary to hunt equids, it should not be ruled out for deer hunting. Unlike selective ambush hunting which would generate profiles dominated by prime adults [5, 129], Bunn and Gurtov [127] point out that non-selective ambush hunting would generate catastrophic mortality profiles. Ethnoarchaeological observations indicate that other non-selective hunting strategies also generate catastrophic mortality profiles [120, 121, 127, 137]. Middle Mississippi hunters during the Archaic period (1000–1550 BC) produced a catastrophic mortality profile of white-tailed deer in six sites through non-selective hunting by stalking [137]. The hunter groups of the Hadza and Kua generate catastrophic mortality profiles in their camps [127] (Fig 13). Encounter hunting is the style most commonly practiced by the Hadza, although, during the dry season, they usually ambush hunt at night near water sources. In both cases, they use poisoned arrows to kill their prey [120, 121]. The Kua mainly encounter hunt, and kill with bows and arrows, although they are also effective as hunters when stalking or using persistence running and traps [133]. The non-selective mortality profiles of levels K, L and M appear to be the product of non-selective stalking. However, it cannot be completely ruled out that this profile may have been produced by non-selective ambush hunting, as with the profiles generated by other non-human ambush hunters, such as leopards or lions [5].

The results of our analysis indicate that the differences in the mortality profiles within the Abric Romaní sequence are the result of various hunting strategies: 1) Selective hunting strategies were employed for equids in all levels and for cervids in levels E, H, I and Jb. 2) Non-selective hunting strategies were employed for cervids in levels Ja, K, L and M. Prime adults comprise the strongest and healthiest animals in a population. Moreover, the large size of equids implies that they pose a substantial risk to hunters. In non-human predators, this risk is reduced through cooperation, as seen in lions, wolves, spotted hyenas and lycaons. Cooperation makes it possible to hunt prey that considerably exceeds the weight of any individual predator [27]. A trend towards capturing prime and young individuals (juveniles = 2, prime adults = 5, old adult individuals = 3) has also been observed in the levels in which the other large ungulate found in this assemblage, *Bos primigenius*, is present (Ja, L, K and M). The two young individuals are subadult juveniles between 3 and 4 years of age and are very close to reaching the weight of an adult animal. Therefore, cooperative organization of hunting groups would have been an effective way to reduce risk in the capture of these large ungulates [27, 28, 58, 59, 138]. Cervid hunting may have also been cooperative, as with the equids, although it is possible that these animals were brought down by individual hunters, especially the young individuals [24, 54, 65, 135]. Depending on the number of prey animals captured during each hunting episode, equid and deer remains may have accumulated over the course of various occupational events in which Neanderthals hunted. These episodes may have occurred over several months within the same season, during the same occupation events or at different times of the year, but not synchronously, so it seems that simple predation prevails over sequential predation. In the hunting of prime adults (equids and deer), it seems that the selective ambush tactic may have been the most commonly used [1, 5, 24, 28, 128]. Young cervids can be captured in multiple ways—by traps, projectiles or pursuit [24, 54, 138]—but would not have constituted a challenge to hunters. Finally, the catastrophic profiles noted for cervids most likely resulted from encounter hunting by stalking or non-selective ambush hunting [127, 138].

The Neanderthals of the Abric Romaní developed specific hunting strategies based on two characteristics of their prey: the taxa and the age of the individual to be hunted. Each hunting strategy gave rise to specific mortality profiles.

## Conclusions

The mortality profiles identified in the faunal assemblages of the Abric Romaní are useful for studying the hunting strategies of the Neanderthals who occupied the shelter. The ages at death of the equids and cervids reflect marked differences between the two taxa. The equids are always dominated by prime adults, whereas the cervids show great diversity in the sequence. This result indicates that the observed differences in mortality profiles resulted from the use of both selective and non-selective hunting strategies.

Selective hunting strategies were employed for the equids at all levels and for the cervids founds in levels E, H, I and Jb. Non-selective hunting strategies were only employed for the cervids of levels Ja, K, L and M. The capture of equids and cervids was probably carried out by various individuals cooperatively, although individual hunting cannot be ruled out in the case of cervids. Depending on the number of prey animals caught by the hunting groups, it seems that simple predation was the most common tactic. The prime adult may have been captured by means of selective ambush hunting. The selective capture of very young individuals may have been accomplished in multiple ways, without presenting a challenge for the hunters. The tactics that generated the catastrophic profiles may have included encounter hunting by stalking or non-selective ambush hunting. Therefore, different hunting strategies have been identified within the same Neanderthal group, some of which are repeated along the sequence, as seen in the case of the equids, and others that vary according to specific behaviors, as observed in the case of the cervids.

## Supporting information

S1 Table Number of equids and cervids mandibles and maxillae at Abric Romaní, indicating level, archaeological reference, MNE, size, dental series, crown height of teeth and code of wear stage. (XLSX)

## References

[1] Frison GC. The Carter/Kerr-McGee Paleoindian Site: Cultural Resource Management and Archaeological Research. American Antiquity. 1984;49(2):288–314.

[2] Klein RG. Age (Mortality) Profiles as a Means of Distinguishing Hunted Species from Scavenged Ones in Stone Age Archeological Sites. Paleobiology. 1982;8(2):151–8.

[3] Klein RG, Wolf C, Freeman LG, Allwarden K. The use of dental crown heights for constructing age profiles of red deer and similar species in archaeological samples. Journal of Archaeological Science. 1981;8(1):1–31.

[4] Kurtén B. On the variation and population dynamics of fossil and recent mammal populations: Soc. pro Fauna et Flora Fennica; 1953.

[5] Stiner MC. The use of mortality patterns in archaeological studies of hominid predatory adaptations. Journal of Anthropological Archaeology. 1990;9(4):305–51.

[6] Stiner MC. Honor among thieves: A zooarchaeological study of Neanderthal ecology. By Mary C. Stiner. Princeton: Princeton University Press. 1994. 447 pp. ISBN 0-691-03456-7. $69.50 (cloth). American Journal of Physical Anthropology. 1996;99(2):363-.

[7] Voorhies MR. Sampling difficulties in reconstructing late Tertiary mammalian communities. Proceedings North American Paleontological Convention, Part E1969. p. 454–68.

[8] Deevey E. Life Tables for Natural Populations of Animals. The Quarterly Review of Biology. 1947;22(4):283–314. 18921802 10.1086/395888

[9] Pearl R, Miner JR. Experimental Studies on the Duration of Life. XIV. The Comparative Mortality of Certain Lower Organisms. The Quarterly Review of Biology. 1935;10(1):60–79.

[10] Frison GC, Wilson M, Wilson DJ. Fossil bison and artefacts from an early altithermal period arroyo trap in Wyoming. American Antiquity. 1976:28–57.

[11] Reher CA, Frison GC. List of errata for the Vore site, 48CK302, a stratified buffalo jump in the Wyoming Black Hills. Plains Anthropologist. 1980;25(88):xvi–xxxi.

[12] Wilson MC. Population dynamics of the Garnsey Site bison. In: Late Prehistoric Bison Procurement in Southeastern New Mexico: The 1978 Season at the Garnsey Site (LA-18399), edited by John D. Speth and William J. Parry. Technical Report No. 12, Museum of Anthropology, Ann Arbor 1978; 88–129.

[13] Fernandez P, Legendre S. Mortality curves for horses from the Middle Palaeolithic site of Bau de l'Aubesier (Vaucluse, France): methodological, palaeo-ethnological, and palaeo-ecological approaches. Journal of Archaeological Science. 2003;30(12):1577–98.

[14] Levine MA. The use of crown height measurements and eruption-wear sequences to age horse teeth. Ageing and sexing animal bones from archaeological sites. 109: BAR British Series; 1982. p. 223–50.

[15] Brown WAB, Chapman NG. The dentition of red deer (*Cervus elaphus*): a scoring scheme to assess age from wear of the permanent molariform teeth. Journal of Zoology. 1991;224(4):519–36.

[16] Payne S. Kill-off Patterns in Sheep and Goats: the Mandibles from Avvan Kale. Anatolian Studies. 1973;23:281–303.

[17] Payne S. Reference codes for wear states in the mandibular cheek teeth of sheep and goats. Journal of Archaeological Science. 1987;14(6):609–14.

[18] Burke A, Castanet J. Histological Observations of Cementum Growth in Horse Teeth and their Application to Archaeology. Journal of Archaeological Science. 1995;22(4):479–93.

[19] Pike-Tay A. Red deer hunting in the Upper Paleolithic of south-west France: a study in seasonality: BAR Oxford; 1991.

[20] Pike-Tay A, Cabrera Valdés V, Bernaldo de Quirós F. Seasonal variations of the Middle-Upper Paleolithic transition at El Castillo, Cueva Morín and El Pendo (Cantabria, Spain). Journal of Human Evolution. 1999;36(3):283–317. 10074385 10.1006/jhev.1998.0271

[21] Lyman RL. On the Analysis of Vertebrate Mortality Profiles: Sample Size, Mortality Type, and Hunting Pressure. American Antiquity. 1987;52(1):125–42.

[22] Fernandez P, Guadelli J-L, Fosse P. Applying dynamics and comparing life tables for Pleistocene Equidae in anthropic (Bau de l'Aubesier, Combe-Grenal) and carnivore (Fouvent) contexts with modern feral horse populations (Akagera, Pryor Mountain). Journal of Archaeological Science. 2006;33(2):176–84.

[23] Greenfield HJ, Chapman J, Clason AT, Gilbert AS, Hesse B, Milisauskas S. The Origins of Milk and Wool Production in the Old World: A Zooarchaeological Perspective from the Central Balkans [and Comments]. Current Anthropology. 1988;29(4):573–93.

[24] Binford LR. Nunamiut ethnoarchaeology New York, NY: Academic Press; 1978.

[25] Lubinski PM. What is adequate evidence for mass procurement of ungulates in zooarchaeology? Quaternary International. 2013;297:167–75.

[26] Boyd DK, Ream RR, Pletscher DH, Fairchild MW. Prey Taken by Colonizing Wolves and Hunters in the Glacier National Park Area. The Journal of Wildlife Management. 1994;58(2):289–95.

[27] Stiner MC. The antiquity of large game hunting in the Mediterranean Paleolithic: Evidence from mortality patterns. Transitions in prehistory: Papers in honor of Ofer Bar-Yosef. 2009:105–25.

[28] Stiner MC. An Unshakable Middle Paleolithic? Trends versus Conservatism in the Predatory Niche and Their Social Ramifications. Current Anthropology. 2013;54(S8):S288–S304.

[29] Adler DS, Bar-Oz G. Seasonal Patterns of Prey Acquisition and Inter-group Competition During the Middle and Upper Palaeolithic of the Southern Caucasus The Evolution of Hominin Diets: Integrating Approaches to the Study of Palaeolithic Subsistence. Dordrecht: Springer Netherlands; 2009 p. 127–40.

[30] Blasco R, Peris JF. Middle Pleistocene bird consumption at Level XI of Bolomor Cave (Valencia, Spain). Journal of Archaeological Science. 2009;36(10):2213–23.

[31] Bocherens H. Diet and Ecology of Neanderthals: Implications from C and N Isotopes. Neanderthal Lifeways, Subsistence and Technology: One Hundred Fifty Years of Neanderthal Study Dordrecht: Springer Netherlands; 2011 p. 73–85.

[32] Costamagno S, Liliane M, Cédric B, Bernard V, Bruno M. Les Pradelles (Marillac-le-Franc, France): A mousterian reindeer hunting camp? Journal of Anthropological Archaeology. 2006;25(4):466–84.

[33] Daujeard C, Moncel M-H. On Neanderthal subsistence strategies and land use: A regional focus on the Rhone Valley area in southeastern France. Journal of Anthropological Archaeology. 2010;29(3):368–91.

[34] Farizy C, David F, Jaubert J, Eisenmann V. Hommes et bisons du Paléolithique moyen à Mauran (Haute-Garonne). Paris: CNRS Paris; 1994.

[35] Fernandez P. Étude paléontologique et archáozoologique des niveaux d'occupations moustériens au Bau de l'Aubesier (Monieux, Vaucluse): implications biochronologiques et palethnologiques. Lyon: Lyon 1; 2001.

[36] Fernandez P, Faure M, Guérin C, editors. Stratégie de chasse des néanderthaliens du Bau de l'Aubesier (Monieux, Vaucluse): choix et opportunisme1998 1998: APDCA.

[37] Gaudzinski S, Roebroeks W. Adults only. Reindeer hunting at the Middle Palaeolithic site Salzgitter Lebenstedt, Northern Germany. Journal of Human Evolution. 2000;38(4):497–521. doi: 10.1006/jhev.1999.0359 10715194 10.1006/jhev.1999.0359

[38] Hardy BL, Moncel M-H. Neanderthal use of fish, mammals, birds, starchy plants and wood 125–250,000 years ago. PloS one. 2011;6(8):e23768 doi: 10.1371/journal.pone.0023768 21887315 10.1371/journal.pone.0023768 PMC3161061

[39] Hardy K, Buckley S, Collins MJ, Estalrrich A, Brothwell D, Copeland L, et al Neanderthal medics? Evidence for food, cooking, and medicinal plants entrapped in dental calculus. Naturwissenschaften. 2012;99(8):617–26. doi: 10.1007/s00114-012-0942-0 22806252 10.1007/s00114-012-0942-0

[40] Jaubert J, Brugal JP. Contribution à l'étude du mode de vie au Paléolithique moyen: Les chasseurs d'aurochs de La Borde. Les chasseurs d'aurochs de La Borde: un site du Paléolithique moyen (Livernon, Lot)(J Jaubert, M Lorblanchet, H Laville, R Slott-Moller, A Turq, and J-P Brugal, eds) Maison des Sciences de l'Homme, Paris, France (Documents d'Archéologie Française 27). 1990:128–45.

[41] Moncel M H, Daujeard C. The variability of the Middle Palaeolithic on the right bank of the Middle Rhóne Valley (southeast France): Technical traditions or functional choices?. Quaternary International. 2012; 247: 103–124.

[42] Moncel M-H, Daujeard C, Crégut-Nonnoure É, Fernandez P, Faure M, Rin C. L'occupation de la grotte de Saint-Marcel (Ardèche, France) au Paléolithique moyen: straté;gie d'exploitation de l'environnement et type d'occupation de la grotte. L'exemple des couches I, J et J''. Bulletin De La Société Préhistorique Française. 2004;101(2):257–304.

[43] Patou-Mathis M. Neanderthal subsistence behaviours in Europe. International Journal of Osteoarchaeology. 2000;10(5):379–95.

[44] Smith GM. Neanderthal megafaunal exploitation in Western Europe and its dietary implications: A contextual reassessment of La Cotte de St Brelade (Jersey). Journal of Human Evolution. 2015;78:181–201. doi: 10.1016/j.jhevol.2014.10.007 25454779 10.1016/j.jhevol.2014.10.007

[45] Stiner MC. On in situ Attrition and Vertebrate Body Part Profiles. Journal of Archaeological Science. 2002;29(9):979–91.

[46] Thieme H. Lower Palaeolithic hunting spears from Germany. Nature. 1997;385(6619):807 doi: 10.1038/385807a0 9039910 10.1038/385807a0

[47] Voormolen B. Ancient hunters, modern butchers: Schöningen 13II-4, a kill-butchery site dating from the northwest European Lower Palaeolithic. Leiden Faculty of Archaeology, Leiden University; 2008.

[48] Yravedra J, Cobo-Sánchez L. Neanderthal exploitation of ibex and chamois in southwestern Europe. Journal of Human Evolution. 2015;78:12–32. doi: 10.1016/j.jhevol.2014.10.002 25481629 10.1016/j.jhevol.2014.10.002

[49] Richards MP, Taylor G, Steele T, McPherron SP, Soressi M, Jaubert J, et al Isotopic dietary analysis of a Neanderthal and associated fauna from the site of Jonzac (Charente-Maritime), France. Journal of Human Evolution. 2008;55(1):179–85. doi: 10.1016/j.jhevol.2008.02.007 18396318 10.1016/j.jhevol.2008.02.007

[50] Richards MP, Pettitt PB, Trinkaus E, Smith FH, Paunovic M, Karavanic I. Neanderthal diet at Vindija and Neanderthal predation: The evidence from stable isotopes. Proceedings of the National Academy of Sciences. 2000;97(13):7663–6. 10.1073/pnas.120178997 PMC16602 10852955

[51] Fiorenza L, Benazzi S, Henry AG, Salazar-García DC, Blasco R, Picin A, et al To meat or not to meat? New perspectives on Neanderthal ecology. American Journal of Physical Anthropology. 2015;156:43–71. doi: 10.1002/ajpa.22659 25407444 10.1002/ajpa.22659

[52] Steele TE. Variation in mortality profiles of red deer (*Cervus elaphus*) in Middle Palaeolithic assemblages from western Europe. International Journal of Osteoarchaeology. 2004;14(3–4):307–20.

[53] Valensi P, Psathi E. Faunal Exploitation during the Middle Palaeolithic in south-eastern France and north-western Italy. International Journal of Osteoarchaeology. 2004;14(3–4):256–72.

[54] Rendu W. Hunting behaviour and Neanderthal adaptability in the Late Pleistocene site of Pech-de-l'Azé I. Journal of Archaeological Science. 2010;37(8):1798–810.

[55] Gaudzinski S. Wallertheim revisited: A re-analysis of the fauna from the middle Palaeolithic site of Wallertheim (Rheinhessen/Germany). Journal of Archaeological Science. 1995;22(1):51–66.

[56] Yeshurun R, Bar-Oz G, Weinstein-Evron M. Modern hunting behaviour in the early Middle Palaeolithic: Faunal remains from Misliya Cave, Mount Carmel, Israel. Journal of Human Evolution. 2007;53(6):656–77 doi: 10.1016/j.jhevol.2007.05.008 17669471 10.1016/j.jhevol.2007.05.008

[57] Stiner MC, Gopher A, Barkai R. Hearth-side socioeconomics, hunting and paleoecology during the late Lower Palaeolithic at Qesem Cave, Israel. Journal of Human Evolution. 2011;60(2):213–33. doi: 10.1016/j.jhevol.2010.10.006 21146194 10.1016/j.jhevol.2010.10.006

[58] Saladié P, Huguet R, Díez C, Rodríguez-Hidalgo A, Cáceres I, Vallverdú J, et al Carcass transport decisions in *Homo antecessor* subsistence strategies. Journal of Human Evolution. 2011;61(4):425–46. doi: 10.1016/j.jhevol.2011.05.012 21802117 10.1016/j.jhevol.2011.05.012

[59] Rodríguez-Hidalgo A, Saladié P, Ollé A, Carbonell E. Hominin subsistence and site function of TD10.1 bone bed level at Gran Dolina site (Atapuerca) during the late Acheulean. Journal of Quaternary Science. 2015;30(7):679–701.

[60] Domínguez-Rodrigo M, Barba R, Soto E, Sesé C, Santonja M, Pérez-González A, et al Another window to the subsistence of Middle Pleistocene hominins in Europe: A taphonomic study of Cuesta de la Bajada (Teruel, Spain). Quaternary Science Reviews. 2015;126:67–95.

[61] Bunn HT, Pickering TR. Methodological recommendations for ungulate mortality analyses in paleoanthropology. Quaternary Research. 2010;74(3):388–94.

[62] Domínguez-Rodrigo M, Barba R. New estimates of tooth mark and percussion mark frequencies at the FLK Zinj site: the carnivore-hominid-carnivore hypothesis falsified. Journal of Human Evolution. 2006;50(2):170–94. doi: 10.1016/j.jhevol.2005.09.005 16413934 10.1016/j.jhevol.2005.09.005

[63] Stephens DW, Krebs JR. Foraging theory: Princeton University Press; 1986.

[64] Steele DG, Baker BW, Hudson J. Multiple predation: a definitive human hunting strategy. From Bones to Behavior. 1993:9–37.

[65] Driver JC. Social hunting and multiple predation. MASCA Research Papers in Science and Archaeology. 1995;12:23–38.

[66] Lombard M, Phillipson L. Indications of bow and stone-tipped arrow use 64 000 years ago in KwaZulu-Natal, South Africa. Antiquity. 2010;84(325):635–48.

[67] Bischoff JL, Julia R, Mora R. Uranium-series dating of the Mousterian occupation at Abric Romani, Spain. Nature. 1988;332(6159):68–70.

[68] Burjachs F, López-García JM, Allué E, Blain H-A, Rivals F, Bennásar M, et al Palaeoecology of Neanderthals during Dansgaard-Oeschger cycles in northeastern Iberia (Abric Romaní): From regional to global scale. Quaternary International. 2012;247:26–37.

[69] Vallverdú J. Micromorfología de las facies sedimentarias de la Sierra de Atapuerca y del nivel J del Abric Romaní. Implicaciones geoarqueológicas y paleoetnográficas. 2002.

[70] Vallverdú-Poch J, Courty M-A. Microstratigraphic Analysis of Level J Deposits: A Dual Paleoenvironmental-Paleoethnographic Contribution to Palaeolithic Archaeology at the Abric Romaní High Resolution Archaeology and Neanderthal Behaviour: Time and Space in Level J of Abric Romaní (Capellades, Spain). Dordrecht: Springer Netherlands 2012; p. 77–133.

[71] Vallverdú-Poch J, Gómez de Soler B, Vaquero M, Bischoff JL. The Abric Romaní Site and the Capellades Region In: Carbonell E, editor. High Resolution Archaeology and Neanderthal Behaviour: Time and Space in Level J of Abric Romaní (Capellades, Spain). Dordrecht: Springer Netherlands; 2012 p. 19–46.

[72] Allué E, Burjachs F, García A, López-García JM, Bennásar M, Rivals F, et al Neanderthal Landscapes and Their Home Environment: Flora and Fauna Records from Level J In: Carbonell E, editor. High Resolution Archaeology and Neanderthal Behavior: Time and Space in Level J of Abric Romaní (Capellades, Spain). Dordrecht: Springer Netherlands; 2012 p. 135–57.

[73] Carbonell E. Abric Romaní nivell I: Models d'ocupació de curta durada de fa 46.000 anys a la Cinglera del Capelló (Capellades, Anoia, Barcelona): Univeritat Rovira i Virgili, Grup de Recerca d'Autoecologia Humana del Quaternari; 2002.

[74] Castro-Curel Z, Carbonell E. Wood Pseudomorphs from Level I at Abric Romaní, Barcelona, Spain. Journal of Field Archaeology. 1995;22(3):376–84.

[75] Solé A, Allué E, Carbonell E. Hearth-Related Wood Remains from Abric Romaní Layer M (Capellades, Spain). Journal of Anthropological Research. 2014;69(4):535–59.

[76] Chacón MG, Fernández-Laso MC. Modelos de ocupación durante el Paleolítico medio: El nivel K del Abric Romaní (Capellades, Barcelona, España)/Patterns of occupation in the Middle Paleolithic: The Abric Romaní level K (Capellades, Barcelona, Spain). Complutum. 2007;18:47–60.

[77] Martínez K, Rando JM. Organización espacial y de la producción lítica en el desarrollo de las actividades durante ocupaciones del Paleolítico medio: nivel Ja del Abric Romaní (Capellades, Barcelona). En 3º Congresso de Arqueología Peninsular: UTAD; 2000; Vila Real, Portugal: ADECAP; 2000. p. 215–34.

[78] Vaquero M. The history of stones: behavioural inferences and temporal resolution of an archaeological assemblage from the Middle Palaeolithic. Journal of Archaeological Science. 2008;35(12):3178–85.

[79] Vaquero M, Vallverdú J, Rosell J, Pastó I, Allué E. Neandertal Behavior at the Middle Palaeolithic Site of Abric Romané, Capellades, Spain. Journal of Field Archaeology. 2001;28(1–2):93–114.

[80] Vaquero M. Neandertal spatial behavior and social structure: hearth-related assemblages from the Abric Romani Middle Palaeolithic site In: Settlement Dynamics of the Middle Paleolithic and Middle Stone Age; 2004; Tübingen: Kerns Verlag.

[81] Vaquero M, Chacón MG, García-Antón MD, Gómez de Soler B, Martínez K, Cuartero F. Time and space in the formation of lithic assemblages: The example of Abric Romaní Level J. Quaternary International. 2012;247: 162–81.

[82] Chacón MG, Fernández-Laso MC, García-Antón MD, Allué E, editors. Level K and L from Abric Romaní (Barcelona, Spain): procurement resources and territory management in short occupations during the Middle Palaeolithic. Raw material supply areas and food supply areas integrated approach of the behaviours Session WS23; 2007 2007.

[83] Gómez de Soler B. Áreas de captación y estrategias de aprovisionamiento de rocas silíceas en el nivel L del Abric Romaní (Capellades, Barcelona). Tarragona: Universitat Rovira i Virgili; 2007.

[84] Gómez de Soler B. Procedencia Del Aprovisionamiento Lítico Durante el Paleolítico Medio en el Yacimiento del Abric Romaní (Capelladas, Barcelona). Niveles M, Oa y P. Tarragona: Universitat Rovira i Virgili; 2016.

[85] Morant N, García-Antón M D. Estudio de las materias primas líticas del nivel I del Abric Romaní. Paleolítico da península Ibérica. Actas do 3 Congresso do Arqueologia Peninsular. 2000.

[86] Fernández-Laso MC, Rivals F, Rosell J. Intra-site changes in seasonality and their consequences on the faunal assemblages from Abric Romaní (Middle Palaeolithic, Spain) Quaternaire 2010;21/2.

[87] Gabucio MJ, Cáceres I, Rosell J, Saladié P, Vallverdú J. From small bone fragments to Neanderthal activity areas: The case of Level O of the Abric Romaní (Capellades, Barcelona, Spain). Quaternary International. 2014;330:36–51.

[88] Rosell J, Blasco R, Fernández-Laso MC, Vaquero M, Carbonell E. Connecting areas: Faunal refits as a diagnostic element to identify synchronicity in the Abric Romaní archaeological assemblages. Quaternary International. 2012;252:56–67.

[89] Rosell J, Cáceres I, Blasco R, Bennásar M, Bravo P, Campeny G, et al A zooarchaeological contribution to establish occupational patterns at Level J of Abric Romaní (Barcelona, Spain). Quaternary International. 2012;247:69–84.

[90] Chacón MG, Fernández-Laso MC, Rivals F, editors. Comportements des populations néandertaliennes pendant le MIS 3 à l’Abric Romaní: Les niveaux K, L et M. Variabilité ou continuité?. Transitions, ruptures et continuité en Préhistoire, XXVIIe congrès préhistorique de France 2010; Bordeaux-Les Eyzies: Société Préhistorque Française.

[91] Vallverdú J, Allué E, Bischoff JL, Cáceres I, Carbonell E, Cebriá A, et al Short human occupations in the Middle Palaeolithic level I of the Abric Romaní rock-shelter (Capellades, Barcelona, Spain). Journal of Human Evolution. 2005;48(2):157–74. doi: 10.1016/j.jhevol.2004.10.004 15701529 10.1016/j.jhevol.2004.10.004

[92] Vallverdú J, Vaquero M, Cáceres I, Allué E, Rosell J, Saladié P, et al Sleeping Activity Area within the Site Structure of Archaic Human Groups: Evidence from Abric Romaní Level N Combustion Activity Areas. Current Anthropology. 2010;51(1):137–145.

[93] Carbonell E. High Resolution Archaeology and Neanderthal Behavior: Time and Space in Level J of Abric Romaní (Capellades, Spain): Springer Verlag; 2012.

[94] Marín J, Saladié P, Rodríguez-Hidalgo A, Carbonell E. Ungulate carcass transport strategies at the Middle Palaeolithic site of Abric Romaní (Capellades, Spain). Comptes Rendus Palevol. 2017;16(1):103–21.

[95] Carbonell E, Vaquero M. Monogràfic: L'Abric Romaní. 1992.

[96] Fernandez P. De l'estimation de l'âge individuel dentaire au modèle descriptif des structures d'âge des cohortes fossiles: l'exemple des "Equidae" et du time-specific model en contextes paléobiologiques pléistocenes. Bulletin de la Société préhistorique française 2009;106(1):5–14.

[97] Mariezkurrena K, Altuna J. Contribución al conocimiento del desarollo de la dentición y el esqueleto poscraneal de Cervus elaphus. MUNIBE 35: Sociedad de Ciencias Aranzadi; 1983. p. 149–202.

[98] Azorit CE. Guía para la determinación de la edad del ciervo ibérico (*Cervus elaphus hispanicus*) a través de su dentición: revisión metodológica y técnicas de elección. ANALES 2011;24 (1).

[99] Klein RG, Cruz-Uribe K. The analysis of animal bones from archeological sites Chicago University of Chicago Press; 1984.

[100] Steele TE, Weaver TD. The Modified Triangular Graph: A Refined Method for Comparing Mortality Profiles in Archaeological Samples. Journal of Archaeological Science. 2002;29(3):317–22.

[101] Bunn HT, Kroll EM, Ambrose SH, Behrensmeyer AK, Binford LR, Blumenschine RJ, et al Systematic Butchery by Plio/Pleistocene Hominids at Olduvai Gorge, Tanzania [and Comments and Reply]. Current Anthropology. 1986;27(5):431–52.

[102] Binford LR. Faunal remains from Klasies River mouth. 1984.

[103] Lyman RL. Vertebrate taphonomy: Cambridge University Press; 1994.

[104] Discamps E, Costamagno S. Improving mortality profile analysis in zooarchaeology: a revised zoning for ternary diagrams. Journal of Archaeological Science. 2015;58:62–76.

[105] Steele TE, Weaver TD. Refining the Quadratic Crown Height Method of age estimation: do elk teeth wear quadratically with age? Journal of Archaeological Science. 2012;39(7):2329–34.

[106] Carranza J. Ciervo—*Cervus elaphus* Linnaeus, 1758 In: Salvador A, J C, editors. Enciclopedia virtual de los vertebrados. Madrid: Museo Nacional de Ciencias Naturales; 2011

[107] Ferré JS. Manejo de la especie equina: Ediciones Mundi-Prensa; 1996.

[108] Goodloe RB, Warren RJ, Osborn DA, Hall C. Population Characteristics of Feral Horses on Cumberland Island, Georgia and Their Management Implications. The Journal of Wildlife Management. 2000;64(1):114–21.

[109] Lincoln GA, Youngson RW, Short RV. The social and sexual behaviour of the red deer stag. Journal of reproduction and fertility Supplement. 1970;11:Suppl-11. 5266389

[110] Kelly RL. The lifeways of hunter-gatherers: the foraging spectrum: Cambridge University Press; 2013.

[111] Clark CW, Mangel M. The evolutionary advantages of group foraging. Theoretical Population Biology. 1986;30(1):45–75.

[112] Dzięciołowski R. Relations between the age and size of red deer in Poland. Acta theriologica. 1970;15(17):253–68.

[113] Delpech F. Les faunes du Paléolithique supérieur dans le Sud-Ouest de la France: Ed. du Centre Nat. de la Recherche Scientifique; 1983.

[114] Mariezkurrena K, Altuna J. Biometría y dimorfismo sexual en el esqueleto de *Cervus elaphus* würmiense, postwürmiense y actual del Cantábrico. Munibe. 1983;35(3–4):203–46.

[115] Waechter J. Man Before History. London: Elsevier 1976.

[116] Blasco MF. In the Pursuit of Game: The Mousterian Cave Site of Gabasa 1 in the Spanish Pyrenees. Journal of Anthropological Research. 1997;53(2):177–217.

[117] Speth JD, Tchernov E. The Role of Hunting and Scavenging in Neandertal Procurement Strategies Neanderthals and Modern Humans in Western Asia. Boston, MA: Springer US; 2002 p. 223–39.

[118] Marean CW. Hunter–gatherer foraging strategies in tropical grasslands: model building and testing in the East African Middle and Later Stone Age. Journal of Anthropological Archaeology. 1997;16(3):189–225.

[119] Binford L R. Bones: Ancient Men and Modern Myths. New York, Academic Press 1981.

[120] Bunn HT, Bartram LE, Kroll EM. Variability in bone assemblage formation from Hadza hunting, scavenging, and carcass processing. Journal of Anthropological Archaeology. 1988;7(4):412–57.

[121] O'Connell JF, Hawkes K, Jones NB. Hadza hunting, butchering, and bone transport and their archaeological implications. Journal of Anthropological research. 1988;44(2):113–61.

[122] Schoville Benjamin J., Otarola-Castillo Erik. 2014 A model of hunter-gatherer skeletal element transport: The effect of prey body size, carriers, and distance. Journal of Human Evolution. 73: 1–14. doi: 10.1016/j.jhevol.2014.06.004 25059517 10.1016/j.jhevol.2014.06.004

[123] O'Connell JF, Hawkes K, Blurton Jones N. Reanalysis of large mammal body part transport among the Hadza. Journal of Archaeological Science. 1990;17(3):301–16.

[124] Vaquero M, Pastó I. The Definition of Spatial Units in Middle Palaeolithic Sites: The Hearth-Related Assemblages. Journal of Archaeological Science. 2001;28(11):1209–20.

[125] Monahan CM. The Hadza Carcass Transport Debate Revisited and its Archaeological Implications. Journal of Archaeological Science. 1998;25(5):405–24.

[126] Caceres I, Rosell J, Huguet R. Séquence d’utilisation de la biomasse animale dans le gisement de l’Abric Romaní (Barcelone, Espagne). Quaternaire. 1998;9(4):379–83.

[127] Bunn HT, Gurtov AN. Prey mortality profiles indicate that Early Pleistocene Homo at Olduvai was an ambush predator. Quaternary International. 2014;322–323:44–53.

[128] Alvard M, Alcorn JB, Bodmer RE, Hames R, Hill K, Hudson J, et al Intraspecific Prey Choice by Amazonian Hunters [and Comments and Reply]. Current Anthropology. 1995;36(5):789–818.

[129] Bunn HT, Pickering TR. Bovid mortality profiles in paleoecological context falsify hypotheses of endurance running-hunting and passive scavenging by early Pleistocene hominins. Quaternary Research. 2010; 74(3): 395–404.

[130] Speth JD. Middle Paleolithic Large-Mammal Hunting in the Southern Levant. Zooarchaeology and Modern Human Origins: Human Hunting Behaviour during the Later Pleistocene. Dordrecht: Springer Netherlands; 2013 p. 19–43.

[131] Tortosa JEA, Bonilla VV, Ripoll MP, Valle RM, Calatayud PG. Big Game and Small Prey: Paleolithic and Epipaleolithic Economy from Valencia (Spain). Journal of Archaeological Method and Theory. 2002;9(3):215–68.

[132] Varin E. Chevreuil, cerf, sanglier: Etudes et récits d'un chasseur: les Editions de l'Orée; 1980.

[133] Lee RB, Yellen JE. The Dobe-/Du/da environment: Background to a hunting and gathering way of life Harvard University Press; 1976.

[134] Martínez K. Análisis funcional de industrias líticas del pleistoceno superior. El paleolítico medio del Abric Romaní (Capellades, Barcelona) y el paleolítico superior de Üçagizli (Hatay, Turquia) y el Molí del Salt (Vimbodí, Tarragona). Cambios en los patrones funcionales. Tarragona: Universitat Rovira i Virgili; 2008.

[135] Schaller GB. The Serengeti LionUniversity of Chicago Press. Chicago and London. 1972.

[136] Fox MW. The whistling hunters: field studies of the Asiatic wild dog (Cuon alpinus): SUNY Press; 1984.

[137] Smith BD. Middle Mississippi Exploitation of Animal Populations. Anthropological Papers No. 57. Museum of Anthropology, University of Michigan. Ann Arbor. 1975.

[138] White M, Pettitt P, Schreve D. Shoot first, ask questions later: Interpretative narratives of Neanderthal hunting. Quaternary Science Reviews. 2016;140:1–20.

